# How reliably can a material be classified as a nanomaterial? Available particle-sizing techniques at work

**DOI:** 10.1007/s11051-016-3461-7

**Published:** 2016-06-14

**Authors:** Frank Babick, Johannes Mielke, Wendel Wohlleben, Stefan Weigel, Vasile-Dan Hodoroaba

**Affiliations:** Research Group of Mechanical Process Engineering, Institut für Verfahrenstechnik und Umwelttechnik, Technische Universität Dresden (TUD), 01062 Dresden, Germany; Division 6.8 Surface Analysis and Interfacial Chemistry, Bundesanstalt für Materialforschung und -prüfung (BAM), 12205 Berlin, Germany; Department of Material Physics, BASF SE, 67056 Ludwigshafen, Germany; RIKILT – Wageningen UR, 6700 AE Wageningen, The Netherlands; Bundesinstitut für Risikobewertung (BfR), 10589 Berlin, Germany

**Keywords:** Nanomaterial classification, Nanoparticle, Number-weighted median size, Tiered approach, Particle size analysis, Nanometrology, Characterisation techniques

## Abstract

**Abstract:**

Currently established and projected regulatory frameworks require the classification of materials (whether nano or non-nano) as specified by respective definitions, most of which are based on the size of the constituent particles. This brings up the question if currently available techniques for particle size determination are capable of reliably classifying materials that potentially fall under these definitions. In this study, a wide variety of characterisation techniques, including counting, fractionating, and spectroscopic techniques, has been applied to the same set of materials under harmonised conditions. The selected materials comprised well-defined quality control materials (spherical, monodisperse) as well as industrial materials of complex shapes and considerable polydispersity. As a result, each technique could be evaluated with respect to the determination of the number-weighted median size. Recommendations on the most appropriate and efficient use of techniques for different types of material are given.

**Graphical Abstract:**

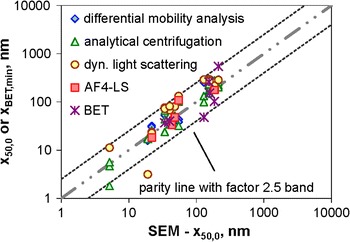

**Electronic supplementary material:**

The online version of this article (doi:10.1007/s11051-016-3461-7) contains supplementary material, which is available to authorized users.

## Introduction

Recent years have seen a tremendous increase in the interest for the development and application of nanomaterials (NMs). Along with this, safety concerns were raised. They were first based on known adverse health effects of particulate airborne matter (fine dust) and second on the experience from other—at that time—new materials with excellent technical properties that after years or even decades of use turned out to have serious adverse effects, e. g., polychlorinated biphenyl (PCB) or asbestos.

**Fig. 1:**
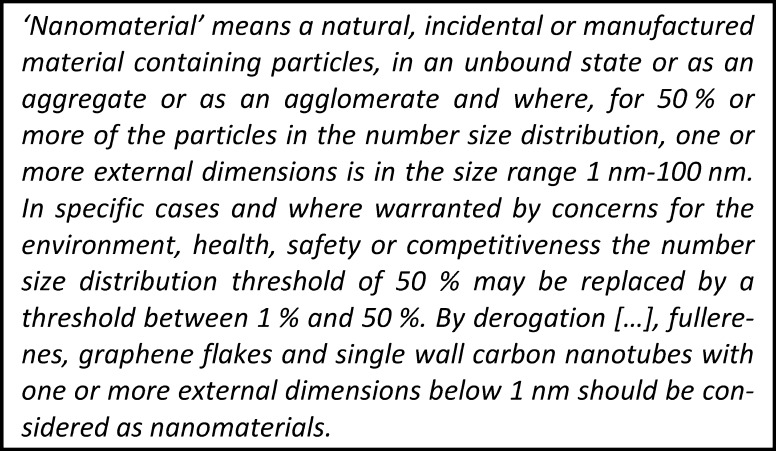
Extract of the recommendation of a definition of NMs by the EC (2011)

As a result, comprehensive efforts into the risk assessment of NMs were initiated and are carried out continuously. Along with this development came the need for a definition of NM for regulatory purposes. The European Commission (EC) recommended a definition in 2011 (2011/696/EU) which focuses on a number-based size distribution (50 % of particles smaller than 100 nm, including constituent particles in agglomerates and aggregates, cf. Fig. 1). The analogous ISO definition (ISO/TS 80004-1) relies on the same size criterion, but does not involve a number-based percentage threshold.

As the EC definition is not restricted to materials intentionally designed to be smaller than 100 nm, it includes virtually all particulate materials with a size distribution into the range below 100 nm, comprising a lot of conventional materials, such as pigments, fillers, additives, etc., and is, thus, relevant for industry, including large as well as small- and medium-sized enterprises.

The meaningful implementation of any NM definition and of the EC definition in particular requires the availability of suited analytical instrumentation and methodologies, yielding consistent and reliable data on the number-weighted size distribution *Q*_0_ for all types and all sizes of particulate material, including highly polydisperse or multimodal ones. Given the broad impact and, thus, need of characterisation for various materials in industry and small- and medium-sized enterprises, the respective analytical technologies also need to be widely available, cost efficient and robust.

The EC recommendation for a definition by a size threshold in number metrics, supported by a threshold in specific surface area as a proxy, was a paradigm change without metrological guidance. Interlaboratory comparisons dedicated to size measurements for nanoparticles as reported in the literature considered number metrics only for techniques with an inherently counting detection principle, such as aerosol quantification by condensation particle counters (Agarwal and Sem 1980; Motzkus et al. 2013; Wang and Flagan 1990), or imaging analysis by electron microscopy (Hodoroaba et al. 2014; Rice et al. 2013; Temmerman et al. 2014a). On the other hand, sizes in volume or surface metrics are critical properties for the performance of commercial particulate materials, and are often part of the product specifications (Brugger 1976), e. g., to differentiate opaque pigments (non-nano) from transparent pigments (nano). Accordingly, techniques for the determination of sizes expressed in volume or surface metrics are well-established, validated by several interlaboratory comparisons (Anderson et al. 2013; Just and Werthmann 1999; Kuchenbecker et al. 2012), and subject to international standardisation (e. g., within ISO/TC 24/SC 4, cf. Table 3 in Appendix 3). However, when referring to these documents care must be taken of the specific applicability of the measurement techniques (MTs) to sub-100 nm size range. In the wake of the EC definition, several authors provided experimental data on the classification by available techniques in volume metrics (Anderson et al. 2013; Gilliland et al. 2014; Just and Werthmann 1999; Kuchenbecker et al. 2012; Wohlleben 2012; Wohlleben and Müller 2014), surface metrics (Hackley and Stefaniak 2013), microscopic counting metrics (Temmerman et al. 2014b; Baalousha et al. 2014), or proposed novel methods (Montes-Burgos et al. 2010).

However, conceptual reviews lamented quite correctly that all these contributions remained very limited in the diversity of test materials, in cross-correlation of techniques with different inherent metrics, or in both—and asked for experimental data to support a self-consistent and widely applicable guidance (Bleeker et al. 2013; Boverhof et al. 2015; Braun et al. 2012; Brown et al. 2013; Gilliland et al. 2014; Linsinger et al. 2012)

Up to date, a systematic evaluation of potentially suited MTs has not been carried out on “real-world” materials, i. e., industrial materials with complex shapes and broad size distributions. Extensive interlaboratory comparison exercises dedicated to the determination of the nanoparticle size distribution have been performed in recent years almost exclusively on rather idealised, “user-friendly” materials, such as spherical, monodisperse and well dispersible silica, polystyrene, or gold nanoparticles, several of them now being certified reference materials (Anderson et al. 2013; Lamberty et al. 2011; Meli et al. 2012; Motzkus et al. 2013; Wang et al. 2007). In some studies, various MTs (mostly TEM, SEM, DLS, AFM, PTA, SAXS, and AC—see list of abbreviations) were taken into account, the corresponding measurement uncertainty budgets were more or less rigorously calculated and the results of the various techniques were compared. Other interlaboratory comparisons were carried out also on well-defined nanoparticle samples, but using only one particular sizing technique in different laboratories, e. g., TEM (Rice et al. 2013), PTA (Hole et al. 2013), or BET (Hackley and Stefaniak 2013). Such systematic studies with a more solid metrological background are a prerequisite for the generation of the standard procedures to be applied to the classification of a (nano)material according to the EC definition using a specific MTs.

The NanoDefine project was set up to support the implementation of the EC definition in all regulatory contexts by an integrated analytical approach. This approach involves the performance evaluation of existing techniques, improvements in instruments and software, development of sample preparation and measurement methods for selected target materials, and provision of guidance by a method manual and an e-tool for selection of the most appropriate (combination of) methods, and classification of materials according to the EC definition. The analytical concept consists of a tiered approach, applying techniques of increasing complexity and complementary measurement principles, but also suggesting adequate procedures for sampling, sample preparation, measurement and data evaluation, as well as plausibility checks and minimum performance requirements.

In this paper, data from a large-scale analytical study on the capability of different MTs to correctly classify particulate materials according to the recommended NM definition are presented. It contains the first European coordinated initiative destined to evaluate experimentally the performance of most of currently applied particle sizing techniques for the characterisation of a broad variety of quality control materials and real-world test materials under harmonised conditions for sample preparation, data analysis, and reporting. As a result, it delivers a unique data set that allows to draw conclusions and to give recommendations on the possible use and limitations of these techniques with respect to the application of the EC definition.

## Design of the analytical study

MTs that are relevant in the context of NM classification are rather diverse with respect to their measurement principles, the type of samples probed, their historical fields of application, and the scientific domains concerned. For this reason, it was neither possible nor meaningful to conduct the analytical study in just one laboratory. Instead, the experimental work was shared among nine participants with long experience and acknowledged expertise for specific MTs. Some MTs were available at more than one participant, and consequently, independent measurements could be used to enhance the validity of the study. In addition, evaluating the “real-world” performance of MTs requires that the particulate samples reflect the real diversity of particulate materials with respect to chemical composition, particle morphology, and size range. For this reason, a rather large set of materials was selected to be representative. In total, the analytical study comprised 174 successful material analyses. Further performed analyses did not yield meaningful results of particle size. For the evaluation of the significance and inter-comparability of such a large and complex data set, it was important to ensure that the single steps of the analytical chain were identical or at least similar among the different laboratories and did not differ principally among the materials. This chain included sampling, primary sample preparation (yielding stock suspensions), secondary sample preparation (for feeding the instrument), instrument preparation (regular qualification, calibration, equilibration, and settings), the actual measurement, data analysis, and reporting all steps.

The following sections reflect the main ideas in selecting materials and MTs, and they describe the concepts of sample preparation, measurement, and data analysis. More details are provided in the Appendices and as supplementary material.

### Particle systems selected

To assess the performance of the selected MTs for the purpose of identifying NMs, a set of 15 different particulate materials was selected and supplied to the participants of the analytical study. This step included procedures for homogenising the original materials and packaging into small units, pre-characterisation with respect to chemical composition and particle morphology, as well as tests for sample homogeneity and short-term stability. The selected materials can be grouped into quality control materials (*QCM*; ISO Guide 30:^2015^), which are intended to qualify the sizing techniques, and representative test materials (*RTM*; ISO/TS 16195:^2013^; Roebben et al. 2013), which are intended to better reflect the measurement challenges proposed by “real-world” materials.

The *QCMs* were composed of individual, i. e., (virtually) non-aggregated, particles of spherical, or sphere-like shape. In addition, the impact of sample preparation was diminished by providing the QCMs as stabilised suspensions. The study used QCMs with rather narrow or with deliberately wide, but well-defined, even trimodal size distributions.

The *RTMs* were commercial powders, for which appropriate dispersion procedures had to be developed before starting the characterisation. The list of RTMs comprised mined and manufactured materials, inorganic and organic ones, materials with amorphous or crystalline phase structure, colour pigments, as well as non-light-absorbing materials. In addition, various types of particle morphology were represented (general irregularly-shaped particulates, needles, and platelets; weakly and strongly bound agglomerates, compact and fractal-like aggregates). Moreover, the study included two material pairs, which referred to different granulometric grades of the same substance.

This selection of RTMs represents the conventional nano and non-nano particulate materials with kiloton-to-megaton production quantities (Keller et al. 2013; Linak et al. 2011; Nowack et al. 2015) for the industry segments of paper and packaging, automotive coatings, plastics in consumer equipment, paints, and anti-caking additives in food and feed. The RTMs do not cover the important classes of reactive or otherwise instable particulates, such as cements and volatile organics, respectively; they further do not cover macroscopic particulate materials, such as polymer granulates and pellets, with constituent particles above 100 µm in volume or surface metrics. Reactive and macroscopic materials pose additional challenges, as they are intended to change their physical and chemical properties just after suspending, dissolution or melting, for which reason the particle size can be severely affected by the milieu (dispersion medium, temperature, pH, etc.). However, one substance (RTM9, basic methacrylate copolymer) represents such an intermediate of relatively larger size.

A brief overview of all QCMs and RTMs is given in Table 1 (Appendix 1). It also provides some information on the presumable polydispersity and NP content, which were all derived from number-weighted distributions of the minimum Feret diameter as measured with a high-resolution SEM (cold field emitter SEM).

**Table 1:** Brief characterisation of quality control materials (QCM) and representative test materials (RTM) employed in this study: substance, morphology, polydispersity with respect to number-weighted distribution of minimum Feret diameter from SEM measurement and cumulative sum at 100 nm

Code	Material	Description	*x*_99,0_/*x*_1,0_ (*x*_max_/*x*_min_)	*Q*_0_ (100 nm) (%)	Misc.
QCM1	Polystyrene	Spherical, monomod., nano	1.7 (1.8)	100	CRM
QCM2	Colloidal SiO_2_	Spherical, monomod., nano	3.0 (5.2)	100	CRM
QCM3	Colloidal Au	Spherical, monomod., nano	1.7 (2.4)	100	
QCM4	Colloidal Ag	Spherical, monomod., nano	3.8 (54)	100	
QCM5	Polystyrene	spher., trimod., nano + sub-µ	12 (14)	88	
QCM6	Colloidal SiO_2_	Spher., trimod., nano + sub-µ	10 (13)	61	
RTM1	BaSO_4_, UF	Compact constituents, aggr., nano	7.1 (21)	99.1	
RTM2	BaSO_4_, fine	Compact constituents, aggr., non-nano	8.5 (16)	7.3	
RTM3	Coated TiO_2_ (Al–Si on rutile core)	Compact constituents, non-nano	4.5 (16)	5	Coating <4 nm
RTM4	CaCO_3_	Cigar-like, non-nano	8.7 (15)	22	Calcite^a^
RTM5	kaolin	Platelets, nano + sub-µ	24 (90)	32	
RTM6	Fumed SiO_2_	Fractal aggregates of nanopart.	2.9 (3.6)	100	
RTM7	Organic pigment Y83, transparent	Needles, aggr., nano	3.5 (4.6)	100	
RTM8	Organic pigment Y83, opaque	Needles, aggr., non-nano	6.1 (7.2)	10	
RTM9	Basic methacrylate copolymer	Compact constituents, micro	14 (40)	14	

^a^Verified by Raman-spectroscopy

### Sample preparation

Sample preparation constitutes a crucial step within the analytical chain, because it determines the state of dispersion which prevails during the measurement. Two phases of sample preparation can be distinguished: a primary phase that provides well-dispersed and stabilised stock suspensions for analysis with different instruments, and a secondary phase that comprises all measures to transfer samples from the stock suspension into the measurement zone. The former is intended to adjust the state of dispersion. In the context of NM characterisation, it aims at the individualisation of the constituent particles or at least at an utmost feasible degree of desagglomeration. In contrast, the secondary sample preparation phase is to conserve the (once achieved) state of dispersion (i. e., to avoid re-agglomeration) when the sample is adapted to the measurement instruments by dilution or addition of various agents (e. g., rheological or colourising agents, electrolytes).

The specific feature of this analytical study is the fact that apart from BET, all characterisation methods are based on suspension samples—even EM analysis. Yet, only the QCMs and RTM6 (fumed silica) were provided as well-dispersed and stabilised suspensions to all participants. These materials did not require sophisticated steps for primary sample preparation; slight agitation (shaking, stirring, and short bath sonication) ensured re-suspension of settled particles and homogenisation of local particle concentration. If the measurement required a dilution, this was realised with filtered, de-ionised water. The spray-DEMA analysis constitutes an exception, because the employed electro-spraying required sample dilution in a particle-free ammonium acetate buffer.

All other RTMs were provided as powder, which meant that the preparation of well-dispersed and stabilised stock suspensions had to be conducted by the participating laboratories. For this purpose, dispersion protocols were developed for each RTM and provided to all partners. These protocols define the wetting agents, stabilising additives, and parameters of dispersion, and were optimised for finest size distributions with cuvAC-turb or DLS. In each case, ultrasonication served as the main technique for desagglomeration, because the stress intensities within cavitational fields are comparatively high (Bałdyga et al. 2009). In addition, participants were advised to control the final state of dispersion via the energy density (Pohl et al. 2004; Sauter et al. 2008). However, implementing the energy density concept in practice has proved to be more challenging than expected. This difficulty occurred, because the participants worked with different types of ultrasonicating disperser (probe sonicators and vial tweeters) and handled different sample quantities (a few millilitres up to 200 mL); an accurate determination of the energy input is particularly challenging for vial tweeters and minute sample quantities. In addition to the differences induced by the local setups used for desagglomeration, also the re-agglomeration in the short time up to the completion of data acquisition is a potential source of differences between laboratories. Yet, we benefited from the fact that some RTMs proved to be well dispersible in the sense that low-energy density suffices to either individualise all constituents or to decompose agglomerates in rigidly bound, hardly dispersible aggregates (e. g., RTM1, RTM3, or RTM9). Regarding the quality of our analytical study, we, therefore, expected for RTM7 and RTM8 a significant impact of sample dispersion on the comparability of measurement results.

Details on sample preparation are provided in Appendix 2.

### Measurement techniques

A critical task in planning this study was the selection of the MTs, since an all-embracing set of MTs would be neither meaningful nor feasible. Hence, different criteria were defined for the selection process, including applicability to the nano-range (<100 nm), ability to directly measure *Q*_0_, availability (for industry, academic world and legal authorities), and accessibility to the project consortium. These criteria emphasise different aspects and were treated as non-exclusive. The final decision on the MT selection was taken after an expert survey.

The following MTs were eventually selected: transmission electron microscopy (TEM), scanning electron microscopy (SEM), single-particle inductively coupled plasma mass-spectrometry (spICP-MS), particle tracking analysis (PTA), differential electrical mobility analysis on sprayed suspensions (spray-DEMA), analytical centrifugation in disc centrifuges with turbidity detector (discAC-turb), analytical centrifugation in cuvette centrifuges with turbidity detector (cuvAC-turb), analytical centrifugation in cuvette centrifuges with refractive index measurement (cuvAC-RI), asymmetric flow field-flow-fractionation with light scattering detection (AF4-LS), dynamic light scattering (DLS), angular light scattering (ALS), small angle X-ray scattering (SAXS), ultrasonic attenuation spectrometry (USSP), and gas adsorption analysis based on the BET method (BET). The main features of these techniques are explained in Table 3 in Appendix 3.

It is clear that the selected MTs could be easily supplemented by other MTs, especially by new developments, which explicitly aim at the characterisation of NMs (e. g., differential surface plasmon microscopy, Sidorenko et al. 2016). In addition, some measurement principles, which can be technically realised in various ways, are only represented by one (frequently used) MT (e. g., AF4-LS as one type of field-flow-fractionation techniques). Last but not least, some MTs were excluded from this study because of their very limited availability (e. g., SANS) or because they are optimised for analytical tasks beyond particle sizing (e. g., AFM). Nevertheless, we believe that our list is a representative collection of available and employed MTs in the field of NM characterisation. It does not only contain established MTs (e.g., AC and ALS), but also relatively new developments (e. g., spICP-MS and PTA).

The selected MTs can be distinguished with respect to the way of particle quantification (by counting, via fractionation, from spectroscopic signals; or by measuring integral signals instead of resolving the size distribution) and with respect to the probed particle property (Bowen 2002; Hassellöv et al. 2008; Hogg 2008). This property may be particle volume or mass, based on particle mobility (including diffusion coefficient, settling velocity, and electric mobility) or related to a scattering pattern. In the case of image analysis, various geometric properties can be determined; in this study, only the minimum Feret diameter was considered (as an estimate of the smallest external dimension).

Based on their technical characteristics, it is possible to express some expectations on the performance of the selected MTs. The first point is that only image analysis offers the chance to directly measure the external dimensions of particles. For isometric and elongated (i. e., needle-like or fibrous) particles, it provides good estimates of the smallest external dimension, but it may be a challenge to do so for flat, platelet-like particles. Scattering patterns, which can be considered as 2D transforms of the 3D morphology, give principally access to all external dimensions, including the smallest one. Yet, this requires that the pattern is measured in high resolution at the relevant scattering angles; for nanoparticles, this is only possible with SAXS (and SANS). In contrast, mass and mobility-based properties cannot resolve the particle morphology, although mobility is affected by it. A typical order of length scale is: largest dimension > hydrodynamic diameter > volume (or mass) equivalent diameter > Stokes diameter > VSSA equivalent diameter > smallest dimension (Appendix 4). For particle aggregates, mass and mobility are always affected by the aggregates outer dimension, but also by the internal aggregate structure. In the worst case (for the purpose of NM classification), the corresponding equivalent diameter is close to the diameter of the aggregate’s convex hull; in the best case, they are upper limits of the constituent particles. For fractal aggregates, it was demonstrated both theoretically and experimentally that the volume equivalent and Stokes diameters are considerably smaller than hydrodynamic or any geometric aggregate diameter (Babick et al. 2012a, b).

Another expectation is related to the quantification of size fractions. If particles are not counted, but quantified by physical properties (e. g., by mass, turbidity, or scattering intensity), then signals of coarse particles may outweigh those of the fine ones. Consequently, the minimum size may be overestimated and the quantity of fine size fractions underestimated. Obviously, this problem is particularly relevant for highly polydisperse materials.

An additional aspect with respect to the analytical task defined by the EC definition for NMs is the distinction between MTs that—by measurement principle—determine sum functions *Q*(*x*) of the size distribution and MTs that inherently measure density functions *q*(*x*) [or transformed density functions q*(*x*)]. The former group comprises all counting and some fractionating techniques (e. g., EM and cuvAC), while the latter is mainly formed by the spectroscopic techniques (e. g., DLS; but also discAC-turb). This is relevant, because the requested median value *x*_50,0_ is a characteristic of the sum function.

All these considerations mean that an imaging technique would be first choice for identification of an NM according to the recommended definition. Therefore, electron microscopy (EM) techniques are considered as reference MTs within this study.

### Particle size measurement and data analysis

From a metrological point of view, the experimental programme of this study must be regarded highly ambitious. Not only do the different MTs determine different intrinsic types of quantity, but these different MTs were placed at different institutions (with specific backgrounds in particle characterisation) and thus run by different operators (with varying expertise and individual preferences). To ensure comparability of measurement results under such conditions requires a common strategy on handling samples as well as conducting and analysing measurements. In a strict sense, the final results may be compared only if they are traceable to the same metrological reference and provided with a realistic measurement uncertainty budget. Even for experienced operators, the quantitative evaluation of the whole traceability chain for the materials selected in the present study is a challenging task. In this study, the following measures were implemented (cf. supplementary material):

- protocols for ensuring a uniform and reproducible state of dispersion at measurement (which goes beyond the sample preparation, described above)
- guidelines for ensuring similar and optimum measurement conditions when working with different instruments of an MT (e. g., in the case of spray DEMA or DLS) or measurement principle (e. g., for all AC instruments)
- rules for replicating measurements to estimate method repeatability (i. e., precision)
- set of consistent values for model parameters (e. g., refractive index, cf. supplementary material S.4)
- a template for reporting the measurement data in a harmonised way (i. e., reporting identical parameters of particle size distribution) and the experimental conditions
- request to check the instrument’s performance with reference materials before starting the experimental programme (qualification of the instruments)

Despite these measures, it was not possible to completely exclude variations in the state of dispersion or to conduct the measurements always at the instrument’s optimum settings. In addition, it was not feasible to evaluate the method repeatability and intermediate precision at the same level of sophistication, because the effort of measurement did considerably vary. For instance, the total time expenditure for a DLS measurement is less than 1 h, but may expand to few hours for EM. As far as we were aware of such imperfections, they were considered in the evaluation of the experimental data.

After having conducted the measurements, size distributions were calculated with the conventional instrument software (usually as provided by the instrument’s manufacturer). We deliberately refrained from using specialised high-end research algorithms, which would distort the “real-world” performance of existing MTs. This means, for instance, that for all but the imaging MTs, the particles were considered homogeneous spheres. A few MTs require a manual pre-treatment of the measured data by experienced operators (e. g., for handling of noise or outliers). When such a pre-treatment is part of the usual analysis procedure, it was allowed as long as it followed clear rules (cf. supplementary material S.2). For the purpose of our study, we primarily compared number-weighted size distributions *Q*_0_. This required the conversion of the intrinsically measured size distributions for some of the MTs (cf. Table 3 in Appendix 3), yet instrument software frequently provides size distributions in any type of quantity (TOQ). The conversion into *Q*_0_ may involve a prior smoothing of measured data (e. g., cuvAC) and/or may employ a model for the intrinsic TOQ, which needs additional material properties (e. g., when intrinsically measuring extinction-weighted size distributions *Q*_ext_). The impact of conversion procedures on the MT’s performance is discussed later in “Influence of the characterisation methodology on the quality of measurement data” section).

## Results

Originally, it was intended to analyse each material with each of the MTs. However, in some cases, it was not possible to conduct measurements because of restrictions set by the sample or by the MT: for instance, BET measurements were not possible for the QCMs, which were supplied as dilute suspensions. USSP measurements could be conducted only on a small set of materials, because the relatively large sample quantities required by the employed instruments (approximately 1–5 g particles) were not available. Organic samples cannot be analysed by spICP-MS. Nanoparticulate BaSO_4_ could not be analysed with spICP-MS, because this substance starts dissolving under the extreme dilution required for spICP-MS analysis (in line with the dissociation constant). For some techniques (e. g., ALS and PTA), it was not possible to characterise the finest quality control materials (i. e., QCM2, QCM3, and QCM4), since the MT’s detection limits for these materials are far above 100 nm. In addition, we encountered difficulties during the analysis of a few materials, which were related to sample preparation rather than to technical limitations. For example, when samples were stabilised with surfactants, their spray aerosolisation for DEMA would be impeded due to foaming. All combinations, which did not allow reliable measurements, are indicated as “n.m.” (not measurable) in Table 4 (Appendix 6).

The realised measurement programme remains, nevertheless, significant enough to conclude on the principal performance of the selected MTs for the identification of NMs. This section will first present and summarise the results of the QCMs. In a second step, results of selected RTMs are shown in detail. To focus on the accurate determination of the number-weighted median size *x*_50,0_, only the cumulative functions of the number-weighted size distributions *Q*_0_(*x*) are shown. In this paper, the term “size” either refers to the equivalent diameter specific to the respective MT or to the minimum Feret diameter determined by imaging techniques (i. e., TEM and SEM). Values and graphs for the density functions and for the intrinsically measured size distributions (i. e., weighted in the intrinsic TOQ) are reported in the measurement reports (cf. supplementary material).

Eventually, an overview of all number-weighted median values obtained for each combination of material and MT is given in Table 4 in Appendix 6. When data were provided by two laboratories (instead of typically one), two values are mentioned in the table.

### Quality control materials

The employed QCMs consisted of spherical particles (or at least particles with similar shape) of varying chemical nature (metals, oxide, polystyrene). Four QCMs show a monomodal and relatively narrow size distribution (QCM1, QCM2, QCM3, and QCM4), while two others are polydisperse and multimodal (QCM5 and QCM6). It turned out that the performance of the MTs depended on the group the QCM belongs to. For this reason, the two groups will be separately discussed. Nevertheless, one can also observe some common features.

In general, there is a rough agreement among the intrinsically measured size distributions within a class of characterisation techniques at least for well-stabilised suspensions. This means that *Q*_0_ of EM techniques are mostly in good agreement. In addition, the extinction and volume-weighted size distributions (*Q*_ext_ and *Q*_3_, respectively) of AC techniques agree fairly well, and the same applies to the intensity-weighted size distributions (*Q*_int_) of DLS and AF4-LS.

The intrinsically measured size distributions (e. g., *Q*_int_ or *Q*_ext_) of non-counting MTs, such as DLS or AC sometimes, show coarse particle fractions (even > 100 nm), which virtually “disappear” after conversion into *Q*_0_ (QCM2 and QCM3). Such coarse fractions imply that the particle system had experienced some degree of agglomeration (either in the original sample or after having been fed to the measurement system), yet their detection is typically related to the relatively high sensitivity towards coarse particles and agglomerates. In this regard, conversion can improve the apparent performance of an MT with respect to NM identification, since the EC definition does not ask for the size of agglomerates and aggregates, but for the size of their constituent particles.

On the other hand, one can also observe that conversion into *Q*_0_ may considerably amplify slight differences prevailing in *Q*_ext_ or *Q*_int_, in particular, when these differences refer to the quantification of fine particles (e. g., discAC-turb and cuvAC-turb for QCM3, cf. discussion on data quality).

#### Quality control materials with narrow size distribution

Three of the four QCM materials (QCM1, QCM2, and QCM3) had narrow size distributions in the range of 10 to 100 nm, while the fourth (QCM4) consisted of particles smaller than 10 nm. Graphs of *Q*_0_ for all QCMs are provided in the supplementary material (S.6).

A general observation for these QCMs is that the differences among the results of different MTs or instruments of the same MT increase as the particle size decreases. This even applies to the EM data, which typically agree very well for particle systems with low polydispersity, but significantly deviate from each other for the finest quality control material QCM4. However, this behaviour in the very low size range has no significant consequences on the NM classification according to the EC definition.

A further observation is that some of the MTs did not allow for a characterisation of these QCMs, because their particle size was beyond the accessible measurement range. This applies to ALS and partly to PTA, spray-DEMA, and spICP-MS. PTA could be used only for the coarsest quality control material (QCM1, *x*_50,0_ ≈ 45 nm), for which it generated size distributions close to those of EM. Spray-DEMA was only applicable to particle systems ≥10 nm, which is the lower detection size limit of the instruments employed. In contrast to PTA or spray-DEMA, the limited applicability of spICP-MS depends to a large extent on the material properties. In particular, polymer (organic) particles are not amenable to ICP-MS. Measurement of SiO_2_ particles is hampered by lower sensitivity, high background, and isobaric interferences, so that characterisation in the nano-range is not facilitated; hence, spICP-MS was only applied to the Au-quality control material (QCM3). Last but not least, BET and USSP could not be applied to the QCMs, since the sample amount (mass of particles, total suspension volume) was insufficient, and—in the case of USSP—because particle concentrations were too low.

The following paragraphs briefly address the performance of the selected MTs.

In this study, PTA and spICP-MS are the only counting techniques that do not rely on image analysis. Since both were just applicable to only one of the narrowly distributed QCMs, sound conclusions on their general performance could not be drawn. PTA worked very well for QCM1 (*x*_50,0_ = 50 nm, which is close to EM’s 45 nm), whereas spICP-MS clearly overestimated the size of the Au-quality control material (QCM3; *x*_50,0_ = 26 nm, EM: ≈ 18 nm)—cf. supplementary material and Table 4 in Appendix 6. While the focus of this study lies on the determination of the number-weighted median *x*_50,0_, the measured size distribution may reveal considerably more details on the state of dispersion. This holds particularly true for counting techniques, as they inherently yield highly resolved size distributions.

Among the regarded MTs, spray-DEMA has some unique features. Even though relying on a fractionation of the particle system, it intrinsically measures *Q*_0_. In addition, it is the only aerosol-based MT within the analytical study. However, the aerosolisation of suspended particles coincides with “residual particles”, which are non-intentionally generated particles from dissolved electrolytes or surfactants. These particles typically show an exponential size distribution, which is superposed on the size distribution of the test specimen. Spray-DEMA, therefore, requires the elimination of “residual particles”, a task which can be conducted physically by electro-spraying or during data analysis (assuming that the modes of residual and relevant particles are clearly separated). For the narrowly distributed QCMs, spray-DEMA was in good accordance with EM results (almost perfect agreement for QCM1, 40 % larger size values for QCM2, and 12 % smaller ones for QCM3). Moreover, the differences between the two instruments employed are marginal.

A further group of MTs is formed by the AC techniques, for which generally, only little variation among the results of three techniques (discAC-turb, cuvAC-turb, and cuvAC-RI) is observed (at maximum 4 nm), although the principles of fractionation and quantification are different. Deviations from each other are most pronounced for the finest particle system (QCM4, i. e., 5 nm Ag). A consistent explanation of this behaviour cannot be given; probably a combination of different effects is the reason. Brownian motion is incorporated in the cuvAC-RI data analysis, but it is ignored in classical cuvAC-turb and discAC-turb data analysis. Furthermore, the electromagnetic response of Ag and Au nanoparticles depends on their size (Santillán et al. 2013; Scaffardi and Tocho 2006)—an effect that is not corrected by any of the AC evaluations, which assume that real and imaginary parts of the refractive index are uniform for all particles.

A somewhat unexpected outcome of the QCM characterisation is the similar performances of DLS and AF4-LS. Both agree fairly well with each other (provided that the void peak signal in AF4-LS can be clearly separated, cf. discussion and S.2). In addition, the results of the two different DLS instruments match almost perfectly. However, when compared to EM techniques, *Q*_0_ of both techniques are not highly reliable. While the covered size range is in accordance with EM, the number-weighted median is once underestimated (QCM1 and QCM3), then overestimated (QCM4), and also fits to the EM result (QCM2). This is not really unexpected, since DLS intrinsically weighs size fractions according to their scattering strength, which is roughly proportional to the squared volume within the nano-range and thus matches the trend of insensitivity towards the finest particles. In addition, DLS requires a numerical inversion procedure of the spectral signal (time correlation function), which inevitably introduces some bias on the shape of the distribution function (Stock and Ray 1985). If, for instance, this bias artificially creates a small fraction of fine particles within *Q*_int,_ this may result into a significant overestimation of fine particles within *Q*_0_.

A last MT that was employed to the narrowly distributed QCMs is SAXS, which appears to be very close to the EM results (QCM2 Δ*x*_50,0_ = 2 nm and QCM4 Δ*x*_50,0_ = 1 nm). This MT benefits from its high sensitivity to structures in the nano-range and from the fact that the analysed scattering signals are essentially surface weighted (*Q*_2_), which keeps small possible negative impacts by conversion.

#### Quality control materials with broad size distribution

Two quality control materials, QCM5 and QCM6, possessed a relatively high polydispersity (cf. Table 1 in Appendix 1), but they differed in the details of the size distribution. QCM5 was composed of three narrowly distributed PSL samples, which led to a rather artificially shaped size distribution with distinct peaks in and beyond the nano-range (yet the coarsest fraction at 350 nm is clearly visible in *Q*_3_ only). In contrast, QCM6 was a polydisperse, commercial slurry with three not very distinct size modes. The most interesting feature of both QCMs is the simultaneous presence of nano and non-nano particles (i. e., the existence of size fractions below and above the critical value of 100 nm). The particles in both QCMs were coarser than for narrowly distributed QCMs, for which reason PTA and ALS could be employed now.

**Fig. 2:**
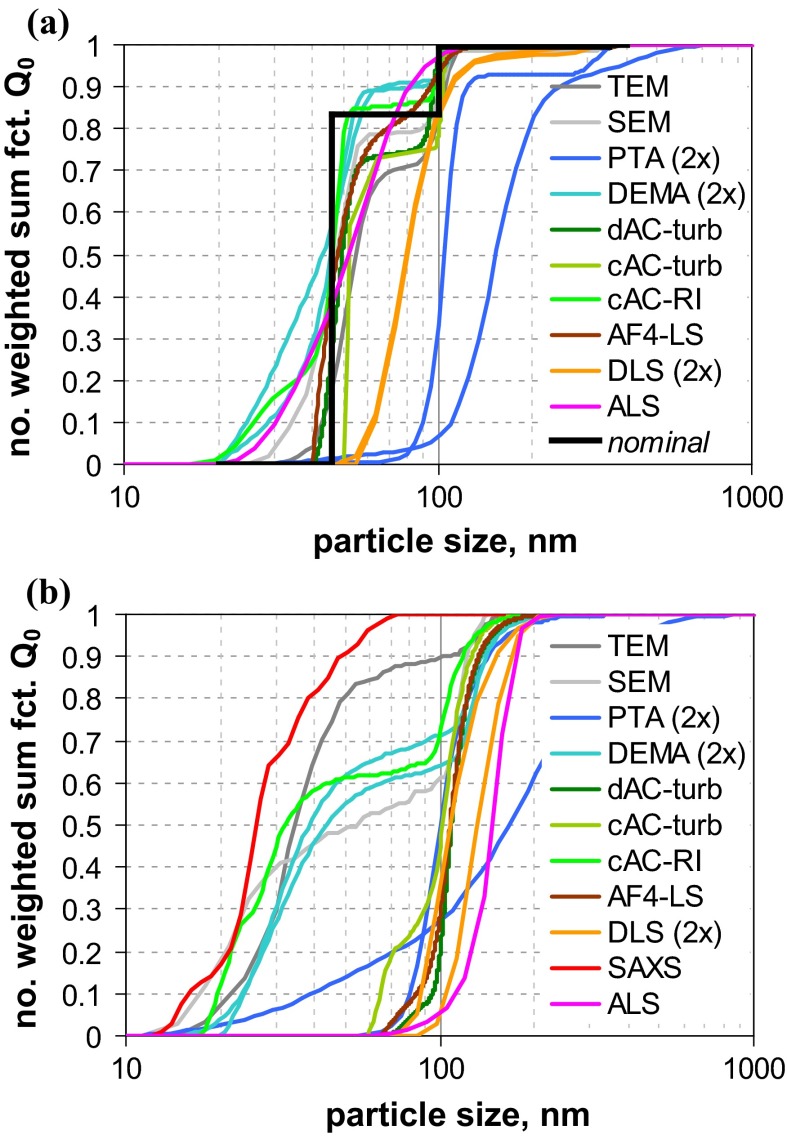
Number-weighted sum functions of **a** QCM5 (trimodal PSL) and **b** QCM6 (trimodal SiO_2_); from measurements with TEM, SEM, PTA (2×), spray-DEMA (2×), discAC-turb, cuvAC-turb, cuvAC-RI, AF4-LS, DLS (2×), ALS and SAXS

A first glance at the results of size analysis (Fig. 2) reveals that the differences among the various MTs are much more pronounced than for the narrowly distributed QCMs. In the case of QCM5 (with clearly separated modes nominally at 46, 100 and 350 nm), most MTs were able to recover the whole size range and even reflect the multimodal shape of the size distribution. However, only few MTs did determine accurately the nominal composition, which was originally defined in terms of mass ratio (cf. Fig. S-19, supplementary material). Best performance with respect to both size and concentration is achieved by AC techniques, followed by EM and AF4-LS.

A different situation prevails for QCM6, where only few MTs indicate a multimodal size distribution. A clear distinction between a significant nanoparticle fraction at around 25 nm and a fraction at around 120 nm is only achieved by EM, spray-DEMA, and cuvAC-RI. The two latter MTs also indicate the presence of a midsize size fraction in the range from 60 to 100 nm with a relative weight of approximately 3 % by number. The existence of this fraction is further confirmed by the other two AC techniques and by AF4-LS, which, however, did not detect the fine mode at 25 nm. The remaining MTs evaluate the particle system as monomodal. While most MTs could not detect the fine NPs in this broadly distributed particle system, SAXS appears insensitive to the coarse particles.

The examples of the two QCMs show that a simple evaluation of a MT’s performance is not possible. Even for the EM techniques, there is no unambiguous picture: While they agree with respect to the size of the different modes, they quantify these modes differently, which is particularly visible for QCM6. This effect can be generally explained by the high sample surface sensitivity, i. e., better visibility of smaller particles onto bigger particles; an effect, which is more relevant for SEM than for TEM. Most small particles situated behind larger particles are invisible for both SEM and TEM. Hence, the observed difference can be attributed to a combination of insufficiently good sample preparation with technique detection capabilities.

Of all the other MTs, only spray-DEMA showed a more or less good agreement with the EM techniques. This is certainly favoured by the fact that both QCMs are well above the lower detection limit of this MT (=10 nm) and that it intrinsically yields *Q*_0_.

All AC techniques recovered the multimodal shape of QCM5 fairly well (with regard to both, size and quantity), but they did not perform uniformly for QCM6. Only cuvAC-RI resolved the trimodal shape and detected the finest particle fraction at around 25 nm. The two other AC techniques, which rely on turbidity measurement, were obviously blind for the fine particles. Nevertheless, both did identify the midsize mode in the range from 60 to 100 nm, which is not seen by most other techniques. Regarding the main features of the distribution functions, there is only minor discrepancy between the turbidity-based AC techniques and AF4-LS.

Unlike with the monomodal QCMs, the performance of DLS and AF4-LS differs for the multimodal QCMs, especially for QCM5. Obviously, the fractionating step by AF4 facilitates the detection of the finest particle mode (at 50 nm), which is not seen by DLS. Yet, for QCM6, both techniques ignore the finest size fraction around 30 nm, which is certainly related to extremely strong dependency of scattering intensity on size in the nano-range (the 30 nm mode of QCM6 scatters approximately 1300 times less than the 100 nm mode; whereas for QCM5, the finest mode of 45 nm scatters approximately 120 times less than the 100 nm mode).

It is interesting to note that ALS performs similarly as AF4-LS. That is, it agrees rather well with EM results for the trimodal QCM5, but clearly ignores the 30 nm mode of QCM6. For this QCM, the number-weighted median is among the coarsest ones.

The largest deviation from EM results of QCM6 is observed for one of the PTA instruments. In the case of QCM5, both PTA instruments underestimated the NP content and clearly failed to classify this material as an NM.

While the “optical” MTs (i. e., DLS, ALS, and PTA) are prone to underestimate the amount of NPs and, thus, to overestimate the number-weighted median *x*_50,0_, the opposite behaviour is demonstrated for SAXS. While this MT performed rather well for the monomodal QCMs, it clearly underestimated the maximum particle size for QCM6 (73 nm as compared to ≈150 nm by EM). This is related to the MT’s lacking sensitivity for particles well above 100 nm. Regarding *x*_50,0_ of this QCM, the impact is rather marginal. However, the deviations may become large for size distributions with increased polydispersity (maximum particle size at 1 µm or above) and generally for all non-nanomaterials.

#### Conclusions on quality control materials

The results of the QCMs have shown that an MT’s performance depends on material, mean particle size, width of the size distribution, and shape of the distribution function. In general, we can state that the size determination of the various MTs is rather reliable for monomodal particle systems with low polydispersity, i. e., all *Q*_0_ are consistent with the results of EM (the difference with respect to *x*_50,0_ is less than 20 % for almost all MTs). This holds true, as long as the particle size falls into the respective measurement range. Several MTs have a lower detection limit well above 1 nm (e. g., spICP-MS, PTA, ALS, and AF4-LS), which restricts their applicability and reduces their general reliability regarding the quantification of nanoparticle fractions. In principle, one should also regard the upper size limits. Yet, for the QCMs (maximum size approximately 350 nm for QCM5), this was just relevant for SAXS, where the upper size limit is approximately 100 nm for the conventional SAXS instrumentation. The QCM analysis has also illustrated the existence of further applications limits, which refer to minimum values for the concentration of suspended particles, sample volume, or total particle mass. Such limits are particularly relevant for BET and USSP, which both could not be applied to the suspension QCMs selected here; however, powders of certified reference materials for BET are available (cf. free data base COMAR, www.comar.bam.de/en/).

Most non-counting MTs are seriously challenged by highly polydisperse samples, because the sensitivity towards a given particle fraction typically increases with size (e. g., a nanoparticle’s contribution to the RI-increment is proportional to its volume, while its scattering intensity is proportional to the squared volume). As a result, the quantity of fine particles is typically underestimated, and the resulting median particle size is too large. In principle, this effect should be more emphasised for spectroscopic MTs (DLS and ALS) than for fractionating MTs (e. g., AC and AF4-LS); this hypothesis could be partially confirmed (QCM5). However, results on QCM6 (multimodal SiO_2_ suspension, i. e., particles with low optical contrast) indicate that the a priori superior performance of fractionating MTs still requires that the measurement signals of the fine particles are sufficiently high for detection. In other terms, the performance of an MT with respect to the measurement of *Q*_0_ is affected by the way of quantification (via fractionation or from spectroscopic signals), as well as by its intrinsic TOQ (proportionality to number, surface, volume, squared volume, etc.).

Last but not least, it should also be noted that even the reference MTs, i. e., TEM and SEM, did not produce unambiguous results for the multimodal QCMs. While their results agree in size range and modal size values, they differed considerably with respect to the quantity of the size modes due to the reasons described above. In general, this may result in a tremendous error of the number-weighted median, which is critical to the context of the recommended NM definition. It is unlikely that the differing results are related to the image analysis, because the QCMs consist of well-stabilised, isolated, and spherical particles. Instead the example points to the most difficult aspect of EM analysis, the preparation of a representative sample of the particle system, while avoiding clustering of the particles upon deposition to ensure that all particles have the same probability of contributing to the measured PSD.

### Representative test materials

Beside the QCMs, the analytical programme comprised nine RTMs, which were prepared using commercial powders consisting of non-spherical, frequently aggregated particles at several size scales (cf. Table 1 in Appendix 1). This section presents the results of four of these materials in more detail; those of the remaining materials are presented in the supplementary material (S.6). An overview of the number-weighted median values of all RTMs as measured with all the MTs in this study is found in Table 4 in Appendix 6.

**Fig. 3:**
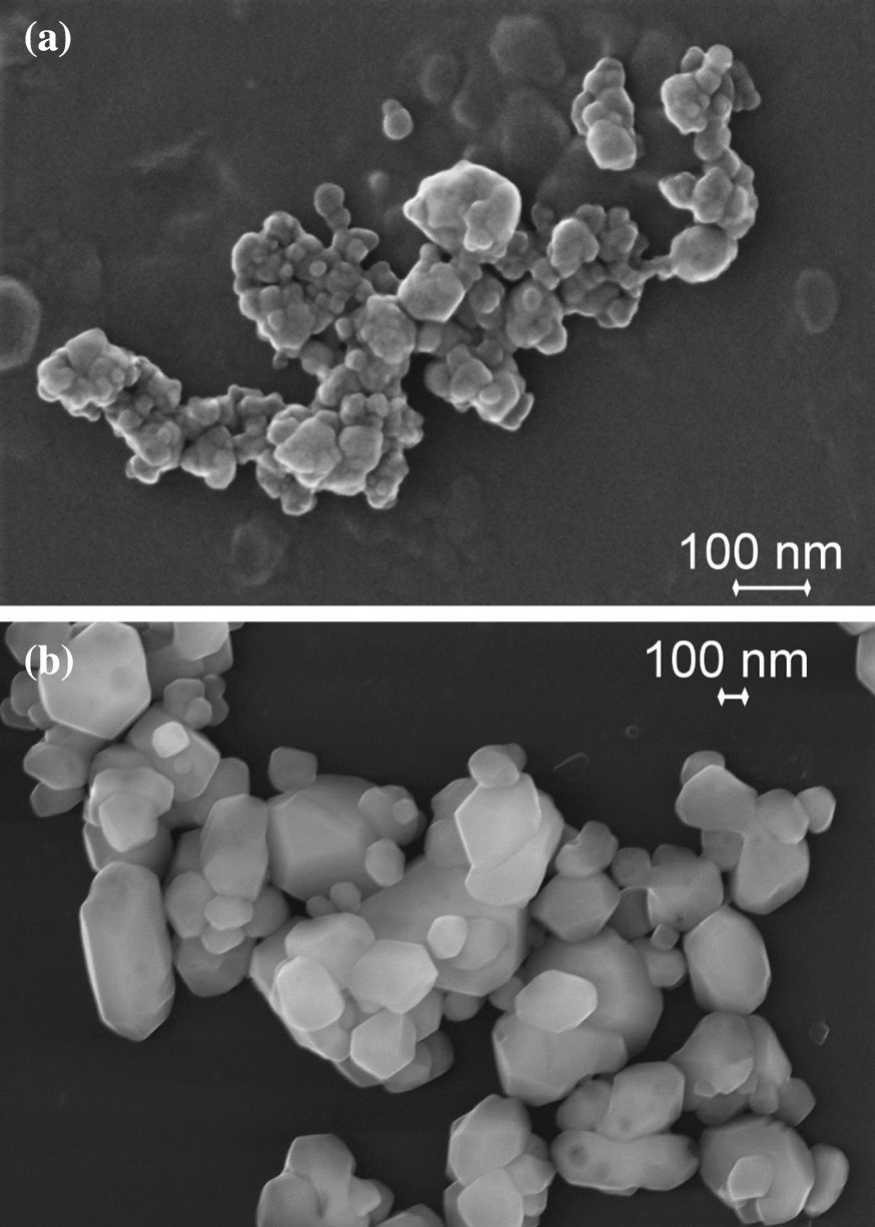
SEM images of **a** RTM1 (BaSO_4_, ultrafine grade) and **b** RTM2 (BaSO_4_, fine grade)

At first, the results of samples RTM1 and RTM2 are discussed. Both are BaSO_4_ powders; yet, they differ in size. In each case, the powder consists of particle aggregates, with constituent particles of compact shape (Fig. 3). Preliminary investigations on the right dispersion procedure implied that a virtually complete disintegration of particle aggregates was possible for the “fine” BaSO_4_ sample RTM2, whereas for the “ultrafine” sample RTM1, only a reproducible state of aggregation was achieved.

**Fig. 4:**
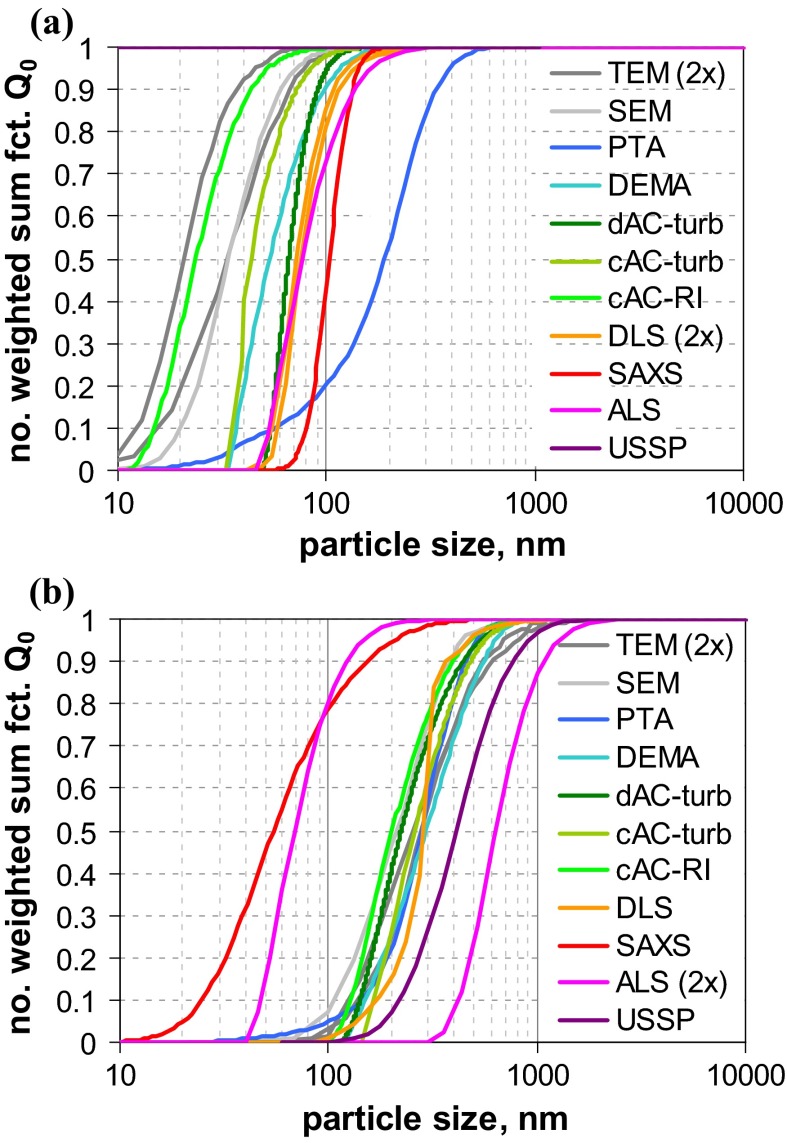
Number-weighted sum functions of **a** RTM1 (ultrafine BaSO_4_) and **b** RTM2 (fine BaSO_4_, right); from measurements with TEM (2×), SEM, PTA, spray-DEMA, discAC-turb, cuvAC-turb, cuvAC-RI, DLS, SAXS, ALS, and USSP

*Q*_0_ of both materials are shown in Fig. 4. For material RTM1, there are obviously considerable variations among all MTs, with EM techniques being among those with the finest size distribution (*x*_50,0_ ≈ 30 nm). EM should deliver the smallest median size for disperse systems that consists of particle aggregates. In detail, one observes a high similarity between DLS and ALS results (*x*_50,0_ ≈ 75 nm) and that the two DLS curves are almost identical. Spray-DEMA yields a slightly smaller result (*x*_50,0_ ≈ 53 nm), while the AC techniques are rather inconsistent (cuvAC-RI *x*_50,0_ ≈ 24 nm, cuvAC-turb *x*_50,0_ ≈ 48 nm, and discAC-turb *x*_50,0_ ≈ 66 nm). Surprisingly, SAXS virtually ignores particles below 70 nm and yields a relatively large value of the number-weighted median (*x*_50,0_ ≈ 103 nm). The coarsest distribution function is obtained with PTA (*x*_50,0_ ≈ 200 nm), even though the presence of nanoparticles is indicated. Based on the measurement principle, PTA should be similar to DLS (since both MTs probe the particle diffusion). The observed discrepancy is probably related to different sensitivities for very fine particles. There is also a *x*_50,0_ result of USSP below 1 nm (cf. Table 4 in Appendix 6) However, it is hardly reliable—probably due to the relatively low particle concentration (≈1 vol%).

Summarising the “real-world” sample RTM1 excellently illustrates the discrepancies among MTs in the case of aggregated systems. EM, which can probe at least a part of the constituent particles, systematically provides smaller values than, e. g., DLS, which is sensitive to hydrodynamic diameter of aggregates; for RTM1, the deviation amounts to a factor of approximately 2.5.

In contrast to RTM1, the other BaSO_4_ material (RTM2) is clearly a non-nano material as almost all MTs indicate. Apart from ALS, USSP, and SAXS, there is a relatively good agreement among all MTs (including PTA), with number-weighted medians in the range of 203 nm (cuvAC-RI) to 293 nm (spray-DEMA), which coincides with the range obtained by EM techniques (212 nm for SEM and 280 nm for TEM). For USSP (*x*_50,0_ ≈ 410 nm), the reliability is again poor, as the particle concentration was at the very low limit of application (1 vol%). The most remarkable feature of the RTM2 analysis is the striking difference between the two ALS results. It should be mentioned that the difference is much smaller in the intrinsically measured volume-weighted size distribution (*Q*_3_) and is instead induced by different concepts of data analysis. The “Discussion of analytical reliability” section provides a more detailed explanation of such effects. A further instructive outcome is the performance of SAXS, which gives the finest size distribution and identifies the material as an NM. This is in line with SAXS performance for QCM6 (Fig. 2b) and reveals a severe shortcoming of this MT with respect to the identification of non-nanomaterials.

Since the two samples RTM1 and RTM2 are different grades of the same substance (BaSO_4_), it is interesting to see to which extent the MTs did reflect the difference in *Q*_0_. In this regard, most MTs (i. e., spray DEMA, AC techniques, and DLS) performed fairly well. However, PTA and ALS clearly failed under this aspect, because the results of the two grades are (partly) quite similar. For SAXS, the evaluation result is even reverse (RTM1 *x*_50,0_ ≈ 103 nm and RTM2 *x*_50,0_ = 54 nm), due to the MT’s insensitivity to coarse particles. An evaluation of USSP was not possible, since the measurement conditions (low particle concentration) could not ensure sufficient reliability. On the other hand, the example implies that this MT may encounter similar limits of application in practice. Finally, attention is drawn to the BET equivalent minimum size (*x*_BET,min_, Table 4 in Appendix 6), which was calculated from volume specific surface area (VSSA) as determined by BET method under the assumption of spherical particles (cf. Appendix 7). The two grades are clearly differentiated, but for RTM1, *x*_BET,min_ is notably, yet not greatly larger than the number-weighted medians of EM (25 %), whereas for RTM2, *x*_BET,min_ is more than twice as large as *x*_50,0_ of EM techniques.

**Fig. 5:**
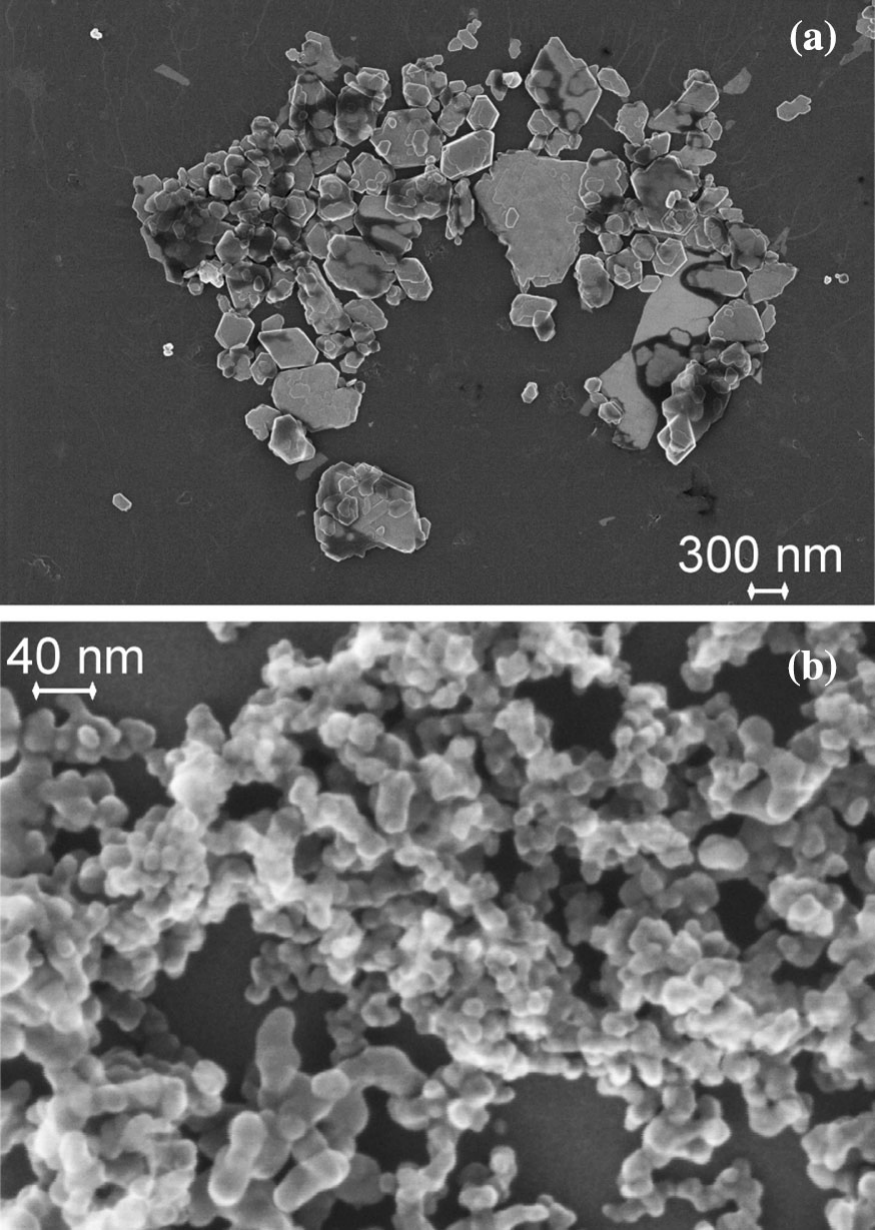
SEM images of **a** RTM5 (kaolin) and **b** RTM6 (fumed SiO_2_)

A second pair of RTMs, which will be examined in detail here, are samples RTM5 (kaolin) and RTM6 (fumed silica), which are well known for the non-spherical morphology of the dispersed phase. The kaolin sample consists of platelet-like particles, which are partially aggregated (Fig. 5a), while fumed silica is composed of highly porous, fractal-like aggregates of nano-sized particles (Fig. 5b). The aggregates of both materials are rather firm and impede a complete dispersion. In particular, for fumed silica, it is known that even intense dispersion by ultrasonication leaves aggregates with dozens or even hundreds of constituent particles (Babick et al. 2012a; Sauter et al. 2008; Wengeler et al. 2006). In contrast, kaolin aggregates are typically formed by just a small number of constituent particles. The major problem of this material is that imaging techniques are biased by the preferential orientation of platelets parallel to the substrate, i. e., the smallest external dimension is typically not accessible to 2D imaging techniques of this type of material.

**Fig. 6:**
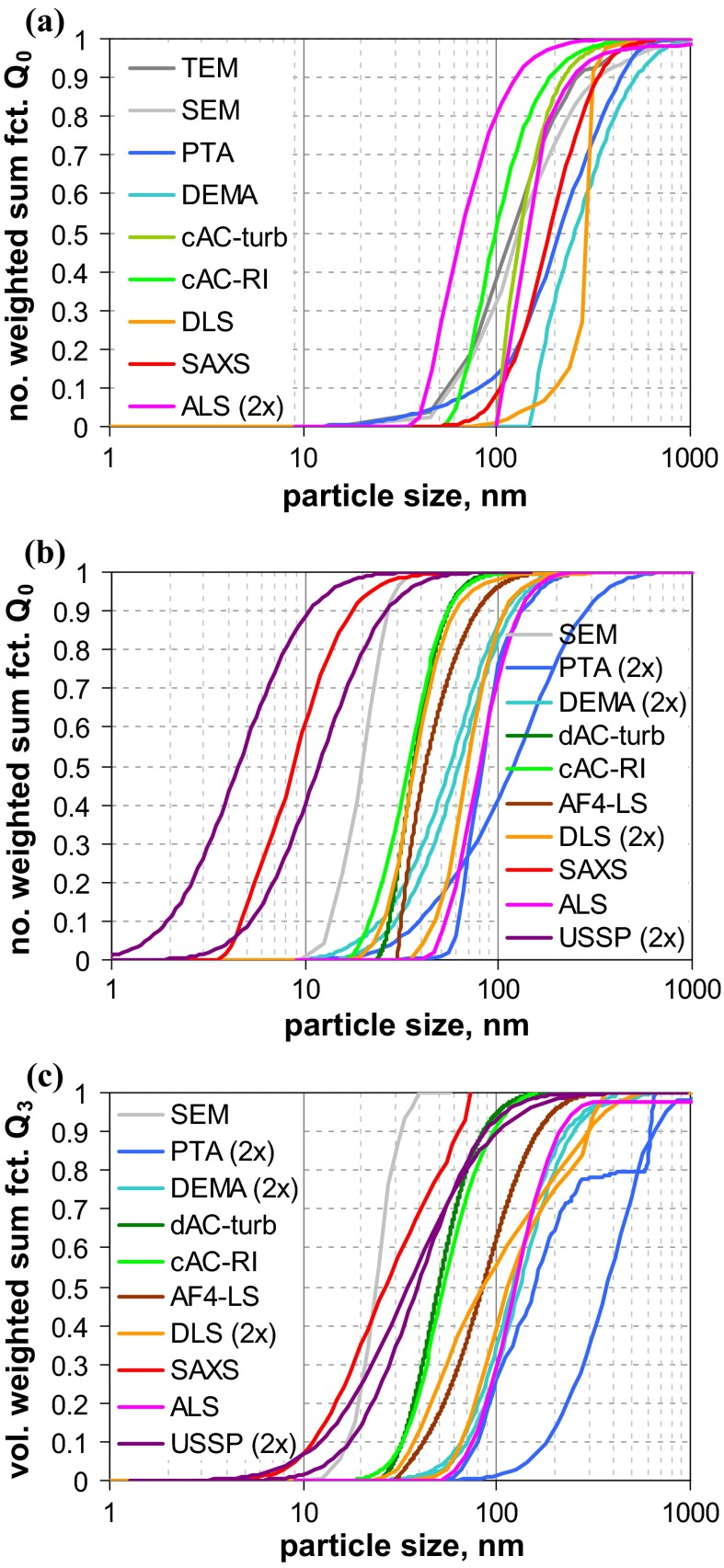
Number-weighted sum functions of **a** RTM5 (kaolin = platelets), and **b** RTM6 (fumed SiO_2_ = aggregates); **c** volume-weighted sum functions of RTM6; from measurements with TEM, SEM, PTA, spray DEMA, discAC-turb, cuvAC-turb, cuvAC-RI, AF4-LS, DLS, SAXS, ALS, and USSP

The graphs of the measured size distributions are presented in Fig. 6. For both materials, a considerable variation among all curves is observed.

The variation seems less pronounced for kaolin, where the results of TEM and SEM (*x*_50,0_ ≈ 125 nm) are similar to those of the AC techniques (*x*_50,0_ ≈ 100 nm … 130 nm). However, one should keep in mind that the EM results are biased toward larger values, since the images emphasise the largest external dimension and conceal the smallest one. The equivalent diameters measured by mobility-based MTs should be lower, as these are affected by all external dimensions, and should deliver a value between thickness and lateral diameter. However, the measured diameters are even larger than those from EM, and specifically, those measured as hydrodynamic diameter are larger (PTA *x*_50,0_ = 212 nm, spray DEMA *x*_50,0_ = 252 nm, and DLS *x*_50,0_ = 290 nm) than those of centrifugation (*x*_50,0_ < 110 nm). The general picture is quite consistent with an aggregated suspension, but the results of AC techniques with turbidity detectors and of DLS should be treated with care, since the conversion into *Q*_0_ assumed spherical shape for the optical models, a shape which is far from reality. The analytical programme included ALS measurements with two different instruments. Their results considerably deviate from each other, which is primarily due to the limited size range of one instrument and further enhanced by conversion. There is also a result obtained by SAXS (*x*_50,0_ = 187 nm), which fits well to the size range covered by the other MTs, but lies beyond the reliable measurement range of this MT. In addition, the kaolin sample RTM5 was characterised by USSP. The number-weighted median is not meaningful (<1 nm, cf. Table 4 in Appendix 6), yet the volume-weighted median seems to be rather plausible (65 nm), which is in accordance with theory that the equivalent diameter of USSP is close to the VSSA equivalent diameter (Babick and Richter 2006). Since the particle concentration was sufficiently high (3 vol%), the results for *Q*_0_ are probably due to the conversion and the simplifying assumption of spherical particles in data analysis.

In summary, most MTs—including TEM and SEM—would classify the material as “non-nano”. Indications that the number-weighted median of the smallest dimension—thickness—is smaller than 100 nm stem from BET (specific surface area, *x*_BET,min_ = 48 nm), ALS (scattering pattern), and USSP (acoustophoretic mobility).

Material RTM6, a fumed silica consisting of fractal-like aggregates, was considered to be the most critical in our study, since (i) the sizes of the constituent particles and the aggregates typically differ by one order of magnitude and (ii) the aggregate porosities are large enough to allow some degree of interstitial flow. The previous studies have already shown that this leads to severe deviations among the various equivalent diameters. This expectation is met by the measurement results (Fig. 6b), which yield number-weighted medians in the range of a few nanometres up to >100 nm. The reference value by SEM (*x*_50,0_ ≈ 20 nm) refers to the constituent particles and is rather consistent with the BET equivalent minimum size (*x*_BET,min_ ≈ 14 nm). SAXS, which also probes the size of the constituent particle, yields a significantly smaller value (*x*_50,0_ ≈ 8 nm). The discrepancy is probably caused by the conversion of SAXS data into *Q*_0_; by volume-weighted medians, the two MTs are fairly close (SEM *x*_50,0_ ≈ 24 nm and SAXS *x*_50,0_ ≈ 26 nm)—in other terms, SAXS probably overestimates the polydispersity. In contrast to SEM, BET, and SAXS, the remaining MTs do not measure the constituent particles, but reflect properties of the aggregates. Apart from USSP and one PTA, they yield number-weighted medians between 37 and 82 nm. The curves imply some systematic differences (e. g., that AC techniques determine finer size distributions than those measuring the hydrodynamic mobility. But also the conversion to number metrics contributes as the main cause of the unreliable *Q*_0_ of USSP. The two PTA instruments yield significantly different results (similar as for QCM5, cf. Fig. 2a). Summing up, most of MTs (apart from one PTA) classify reliably this challenging material as an NM.

### Summary of all experimental data

The previous sections showed examples of results of particle size analysis for different types of particulate materials. The complete set of analysis results is provided in the supplementary material. Below, these size analyses are summarised to discuss the performance of the different MTs after testing them on all selected QCMs as well as RTMs. The summary focuses on the number-weighted median (*x*_50,0_) as the decisive parameter for the classification of particulate materials according to the EC definition. The corresponding values are presented in Table 4 (Appendix 6).

**Fig. 7:**
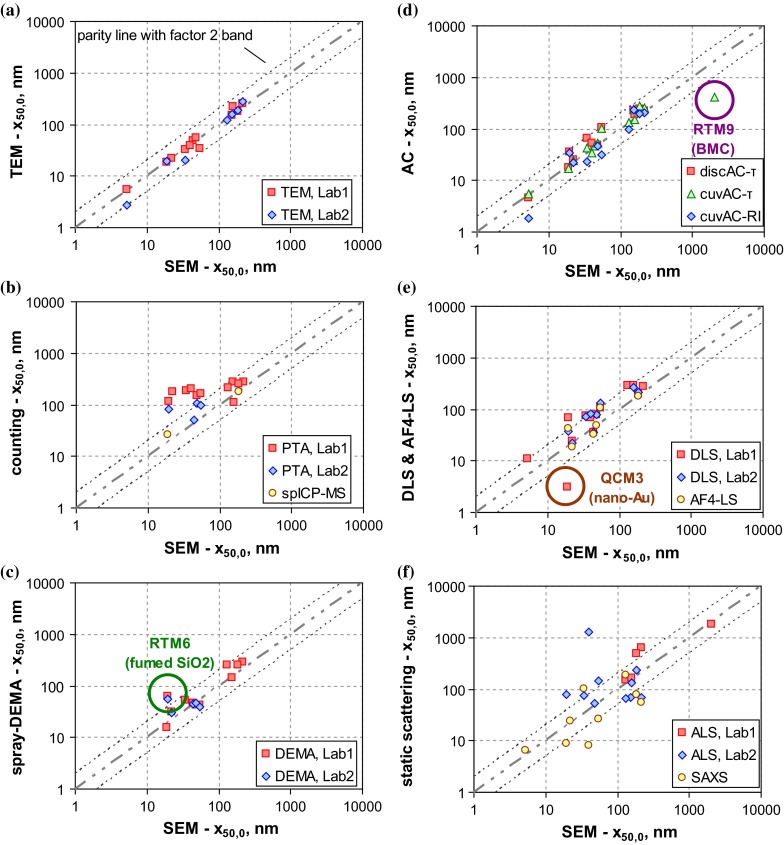
Parity plots of the number weighted medians *x*_50,0_ as determined by the various MTs versus the SEM value (cf. Table 4), lines indicate parity and deviation from parity by a factor of 2; **a** imaging techniques (TEM), **b** non-imaging counting techniques (PTA, spICP-MS), **c** fractionating techniques with a counting detector (spray-DEMA), **d** AC techniques (discAC-turb, cuvAC-turb, cuvAC-RI), **e** mobility-based techniques with a light scattering detector (DLS, AF4-LS), **f** static scattering techniques (ALS, SAXS)

At first, we look at the *SEM* and *TEM* results, because EM techniques had already been identified as most appropriate for the NM classification. This study comprised analyses by two TEM and one SEM instruments. Only the latter was applied to all materials of this study, and hence, its number-weighted median sizes are taken as reference for all other MTs. A parity plot of TEM versus SEM results (Fig. 7a) reveals a good agreement (within 20 % relative deviation) among the three EM results for about half (8 of 15) of the particulate materials. For three further materials (QCM4, QCM6, and RTM1), the ratio between the largest and smallest *x*_50,0_ values lies between 1.5 and 2, and for two other materials, the relative deviation amounts to values between 20 and 50 % and 50 and 100 %. There are manifold sources for such deviations: improper sampling, sample preparation, or particle deposition on the substrate may affect the representativeness of the imaged particles. In addition, the identification of constituent particles and their characterisation are affected by software settings (threshold values, separation algorithms, etc.). In summary, EM techniques provided consistent *x*_50,0_ results for most, yet not all materials. The example of RTM5 (kaolin) has even shown that they might systematically overestimate the smallest external dimension. For this reason, EM results cannot be used alone for the NM classification.

Keeping in mind that SEM can be “blind” to the smallest dimension for few particle morphologies, we subsequently use it for the evaluation of all other MTs.

The outcome for non-imaging counting techniques (PTA, spICP-MS) is shown in Fig. 7b. The graph shows that *PTA* has a general problem to measure particles in the range below 100 nm. This holds particularly true for one of the two instruments (by different manufacturers) employed in this study, which systematically overestimated the number weighted median in the nano-range and even failed to identify NMs as such. The second PTA instrument performed better, especially for the non-aggregated QCMs. However, both instruments struggled with the detection of very fine nanoparticles, for which reason they did not deliver meaningful results for materials QCM2, QCM3, and QCM4. It should be noticed that PTA generally performed better for particle sizes from 100 to 1000 nm. For them, the deviation from SEM results with respect to the number-weighted median (*x*_50,0_) is here between 30 and 80 %.

For *spICP-MS*, only a reduced amount of measurement data is available, as only two materials (QCM3, RTM3) were accessible to size analysis. In both the cases, the MT performed quite well, that is the number-weighted medians are close to the SEM value: 37 % deviation for QCM3 and 2 % for RTM3. However, the lacking applicability to non-metal containing particles (i. e., the majority of QCMs and RTMs) illustrates that this MT cannot serve as a universal tool for the classification of NM.

Figure 7c summarises the results of *spray-DEMA*, which is a representative for fractionating techniques with a counting particle detector. Within this study, one commercial measurement system was independently used by two different laboratories. The figure reveals that their results are highly consistent (difference <20 %, yet for just five materials), and the number-weighted medians deviate from SEM values typically by factors smaller 1.5 (for 8 of 11 materials). Only for the fractal aggregates of RTM6 (fumed silica), spray DEMA overestimated the size by a factor of approximately 3.

A further group of MTs is analytical centrifugation (*AC*), of which this study comprised disc-AC with a turbidity sensor (discAC-turb), cuvette-AC with a turbidity sensor (cuvAC-turb), and cuvette-AC with refractive index measurement (cuvAC-RI). The results of the three AC techniques agree fairly well for most of the materials as shown in Fig. 7d. The maximum variation is ≤50 % for 10 of 14 materials. Large inconsistency (>factor 2) was observed for just three materials (QCM4, QCM6, and RTM1), which was only partly understood. Regarding the conformity with SEM analyses, one observes that for 10 of 15 materials, the number-weighted medians (*x*_50,0_) differ by less than 50 %. There are only two materials, for which they deviate by a factor above 2: QCM3 (which is clearly nano, <10 nm) and RTM9 (which is clearly non-nano, >1 µm).

Figure 7e comprises data of *DLS* and *AF4-LS*. DLS results were obtained by similar instruments in two different laboratories. They are quite consistent with a deviation <25 % for most materials (exception RTM6, for which results differ by a factor of 1.9). However, DLS typically yields more than 50 % larger number-weighted medians than SEM—independent of size range. The exceptions are the monodisperse QCMs 1 and 2 (deviation ≤20 %) and QCM3 (underestimation of size). The factor of overvaluation is less than 2.5 for 13 of 14 materials, but amounts to 3.5 for the fractal-like aggregates of RTM6.

In this study, AF4-LS performed essentially similar to DLS, yet the statement relies on the results of only six materials. Good agreement with SEM (below 25 %) was observed for the monodisperse QCMs and for RTM3 (TiO_2_), which consists of non-aggregated, compact particles with moderate polydispersity, whereas the polydisperse QCM6 and RTM6 were overestimated in size (up to factor of 2.1).

A last graph of this series (Fig. 7f) corresponds to the static scattering techniques *ALS* and *SAXS*. Both techniques defy an unambiguous evaluation and show contradictory results. For ALS, two different instruments were employed, which allows an evaluation of consistency. Obviously, it is rather poor: only in 1 of 5 materials, the results differ by less than 100 % (for RTM1, they differ even by a factor of 9). This is in line with reports by other authors (Kuchenbecker et al. 2012) on the low reliability of ALS data in the submicrometre range. In addition, number-weighted medians by ALS deviate considerably and non-systematically from those by SEM (factor is above 2 for 7 of 11 materials).

Similarly, SAXS also deviates notably from SEM for the majority of materials (well above factor 2). However, good agreement with SEM (<20 %) is found for the monomodal QCMs 2 and 4. For the fractal aggregates of RTM6, the number-weighted median (*x*_50,0_) was underestimated by approximately 50 %, which is probably related to conversion issues (see discussion on Fig. 6), yet also proves that SAXS probed the constituent particles rather than a property of the particle aggregates. Not all SAXS results are really understood. For instance, why for RTM5 (kaolin), *x*_50,0_ by SAXS was approximately three times the BET equivalent minimum size, or why the number-weighted median of RTM7 was five times smaller than that of EM techniques. In total, both MTs are not appropriate for the classification of NM. However, SAXS—unlike ALS—is not yet really well-explored and the MT is still experiencing significant developments.

The graphical summary of Fig. 7 does not include the only acoustical MT in this study, *USSP*, which actually has the principal advantage of covering a broad size range (10 nm to 100 µm), but requires a relatively high minimum particle concentration for reliable measurements. The latter proved to be a critical practical aspect in this study, because sample volumes and particle concentrations had to be kept low to ensure homogeneity among all samples sent to the participants. In this regard, the study could not accurately reflect the MT’s performance, whereas in industrial practice, the sample volume may be of minor relevance. The USSP showed rather interesting results for *Q*_3_ of RTM5 (kaolin with platelet-like particles) and RTM6 (fumed silica), because it was similar to the BET and SEM values, respectively. However, after conversion into *Q*_0,_ the physical plausibility was lost, which shows a need to improve data analysis.

The parity plots of Fig. 7 are a simple and comprehensible way of evaluating the performance of selected MTs with respect to internal consistency and inter-comparability. Our study clearly showed the practical capabilities and limitations of measuring the number-weighted median size (*x*_50,0_) of the constituent particles of particulate materials. Even EM techniques cannot per se claim high accuracy and reliability, although the results are quite consistent for most materials. The parity plots also indicate deficiencies of PTA, ALS, and SAXS in measuring the number-weighted median size (*x*_50,0_) over the particularly interesting size range of 10–1000 nm, which constitutes a severe obstacle for the applicability to NM classification. The graphs also show that all non-counting MTs that are based on the hydrodynamic mobility (i. e., spray DEMA, AC techniques, AF4-LS, and DLS) performed similarly with respect to the *x*_50,0_ determination. This behaviour was already noticed in the previous sections when discussing the shape of *Q*_0_. Such an outcome was not really expected because of the considerable differences in equivalent diameter and intrinsic TOQ. Yet, only in a few cases, these differences are relevant. This refers to particle aggregates with high porosity, for which AC techniques determine finer size distributions, and to materials with high polydispersity, for which spray-DEMA may be more reliable for the fine particle fractions than the others (cf. QCM6, Fig. 2). AC techniques, AF4-LS and DLS require the conversion of *Q*_int_, *Q*_ext,_ or *Q*_3_ into *Q*_0_. This conversion is the most critical step of these techniques, since it amplifies noise, artefact modes, etc. In some cases, this may eventually lead to a mis-classification of the substance (see “Discussion of analytical reliability” section). For DLS, this effect is expected to be even more pronounced, since the conversion bias adds to that of inverting the spectral signal. Although this holds generally true, it was not particularly relevant in this study. Hence, all four mobility-based MTs (spray DEMA, AC techniques, AF4-LS, and DLS) may serve as screening techniques for the classification of real-world particulate materials, even though the performances for RTM6 (fumed SiO_2_) and RTM9 (methacrylate) were partly rather poor. When these materials are excluded from the study, we can show that almost all values of the number-weighted median (*x*_50,0_) agree with the corresponding value of SEM by a factor of 2.5 (cf. Table 4 in Appendix 6). That means, when one of these techniques finds number-weighted medians (*x*_50,0_) above 250 nm, this implies for most materials that they are not an NM according to the EC definition. Similarly, a *x*_50,0_ value below 40 nm strongly indicates that the material is an NM. The borderline region, in which results from different laboratories may not agree, is narrower (factor 2) if MTs are restricted to EM, spray DEMA, and AC, and even narrower (factor 1.5) if MTs are restricted to EM.

Likewise—and an important proposition in the application of the EC definition—the volume, extinction, or intensity-weighted medians, which are intrinsically measured by AC, DLS, or AF4-LS), classify a material as an NM if they are smaller than 100 nm (i. e., a first evaluation of materials is possible without the need of conversion).

Last but not least, the study also employed gas adsorption measurements according to the *BET* method for estimating the VSSA and its corresponding mean value of the smallest particle dimension. This mean size is generally in agreement with EM techniques within a factor 2.5, which is the same tolerance range as found for the non-counting, mobility-based techniques (cf. Table 4 in Appendix 6). However, BET results may be misleading for materials with internal or coating microporosity (RTM3 in Table 4 in Appendix 6). In the borderline region close to the 100 nm cut-off, BET requires confirmation by a second MT. However, the BET analysis facilitates an estimation of the size of constituent particles and may even help to correct biased data from EM techniques, as evidenced by the case of platelet particles (RTM5 in Table 4 in Appendix 6). Beside the access to the smallest dimension, the advantage of BET is that no dispersion protocol is required, so that artefacts are avoided for hydrophobic or soluble NMs (RTM7 and RTM9, respectively, in Table 4 in Appendix 6).

Hitherto, this summary considered the general deviation among the number-weighted median values *x*_50,0_ of the selected MTs. A more specific issue is the reliability of material classification according to the EC definition (i. e., whether *x*_50,0_ is smaller or larger than 100 nm). Due to the absence of validated and universally accepted NM tests, we better ask for the conformity with SEM results concerning the classification as an NM (TEM and SEM agree in this classification for all QCMs and RTMs). This kind of data evaluation is visualised by a colour code in Table 4 (cf. Appendix 6). A first glance on the table shows that the conformity to SEM results is quite frequently achieved, despite the fact that the majority of employed MTs do neither probe the external dimension of the (constituent) particles, nor they intrinsically determine *Q*_0_. One explanation of this surprisingly good outcome may be that most materials employed are either clearly in the nano-range (*x*_50,0_ below 50 nm even with respect to aggregates) or are well above it (*x*_50,0_ above 200 nm). There are only few materials, for which SEM indicates significant fractions of nano and non-nanoparticles (in particular, QCM6, RTM4, and RTM5, cf. Table 1 in Appendix 1). Indeed, most deviations from the SEM classification are observed for QCM6, which has a nanoparticle content of 61 % (by number) according to SEM (cf. Fig. 2b).

A further outcome of this evaluation is that most MTs employed could reliably differentiate between the nano and non-nano grades of BaSO_4_ (RTM1 and RTM2) and the organic pigment (RTM7 and RTM8). This holds true for the three AC techniques, DLS and BET, whereas PTA, ALS, and SAXS yielded contradictory results. These differences in performances are in accordance with the conclusions from the parity plots (Fig. 7) and will be adequately considered in the “Discussion of analytical reliability” section.

In general, we found that if the materials median size is not too close to 100 nm (outside the range 50–150 nm), a material classification according to the EC definition which is based on values in Table 4 in Appendix 6 led to (i) very few false negatives, i. e., the results did not classify a NM as non-NMs and (ii) very few false positives, i. e., only few results classified a non-NMs as NM.

## Discussion of analytical reliability

The question for the reliability of NM classification with currently available MTs may provoke quick and general answers. Yet, in practice, the performances of MTs depend also—to a more or a lesser extent—on the specific material, the quality of measurement procedures (incl. sample preparation), and the appropriateness of data analysis. For this reason, it is not sufficient to select appropriate MTs for the identification of NMs, but incorporate these techniques into characterisation methods, which define all steps from sampling to data analysis. A further aspect is the precision with which the measurement results are obtained. Consequently, our discussion will, therefore, first look to the general data quality, and subsequently discuss the potential impact of the steps of analysis on measurement result, before it evaluates the outcome of this study.

### Estimation of measurement uncertainty within this study

The evaluation of the accuracy of the results relies on the assessment of its two components, precision—including repeatability, intermediate precision and reproducibility—and trueness. While precision is in principle easily to evaluate for all the employed MTs, but practically time-consuming, the evaluation of trueness, i. e., of the deviation of the result obtained from its true value, is generally a challenging task necessitating considerable expert knowledge of the individual technique, instrument, and software. An overview of the relative repeatability and intermediate precision attained in this analytical study can be seen in Table 6 (Appendix 8). Note the small values (below 5 %) corresponding to the majority of techniques and materials. The estimation of trueness has been performed in this study partly by means of using the QCMs, i. e., samples with well-known particle size distribution. Further sources of measurement uncertainties generating systematic (bias) deviations have been already addressed in the previous sections on a more material related basis and are discussed qualitatively in the next sections on a rather more methodical basis (robustness, sample preparation, data reduction, etc.).

Without a rigorous evaluation of the measurement uncertainty budgets associated to the results obtained in this study, a direct comparison of all the results generated by different techniques is in fact hardly possible. Nevertheless, the comparison was done taking the results obtained by EM as a reference and discussing the potential sources of uncertainties for each technique in part in a semi-quantitative way. For the EM systematic, metrological studies (Hodoroaba et al. 2014; Motzkus et al. 2013; Rice et al. 2013; Temmerman et al. 2014a) have been recently carried out by various research groups with the purpose of estimation of the measurement uncertainties related to model samples (such as the QCMs in this study). However, with a few exceptions (Braun et al. 2011), for most of the other MTs, the metrological basis for application in the size range relevant for this analytical study is simply missing.

The present study does not claim to be a metrologically rigorous interlaboratory comparison of a large variety of complex, real-world materials with almost all available sizing techniques. After this unique systematic analytical study, the NanoDefine project has planned to group techniques and materials to proceed further with intra- and interlaboratory validation which may even result in standardised methods.

A particular situation exists for counting techniques, for which the number of analysed particles is typically much lower than for fractionating or spectroscopic techniques (e. g., assuming a typical scattering volume of 10^6^ µm^3^ in DLS (Willemse et al. 1997) means for a suspension with 0.01 vol% 100 nm particles that at each instant approximately 2 × 10^5^ particles contribute to the signal). It is, therefore, necessary to understand, to which degree the measured *Q*_0_ is affected by the amount of sample probed.

We examined this effect for the EM techniques, since they are considered most appropriate for the implementation of NM classification. Even if the imaged particles can be considered representative, they will never perfectly reflect the size distribution in the original particle system. The stochastic event of collecting particles of a certain size in the analysed sample can be described by Poisson statistics. To examine the impact of such stochastic fluctuations on the measured size distribution, the set of acquired micrographs was divided in random subsets of different sizes (i. e., numbers of considered particles). The size distributions of these subsets were formed without binning, as the primary focus lies on the number-weighted median size.

**Fig. 8:**
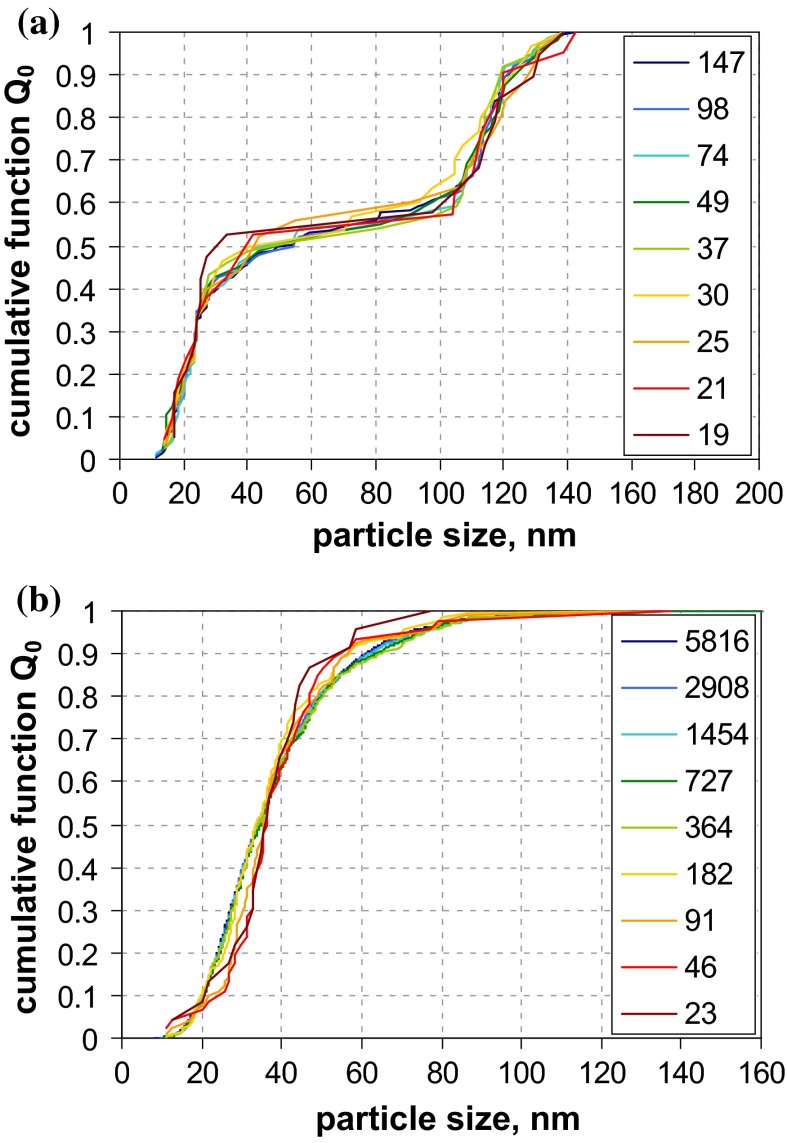
Impact of no. of evaluated particles on number-weighted size (*x*_Feret,min_) distribution by SEM; for **a** QCM6 and **b** RTM1

**Fig. 9:**
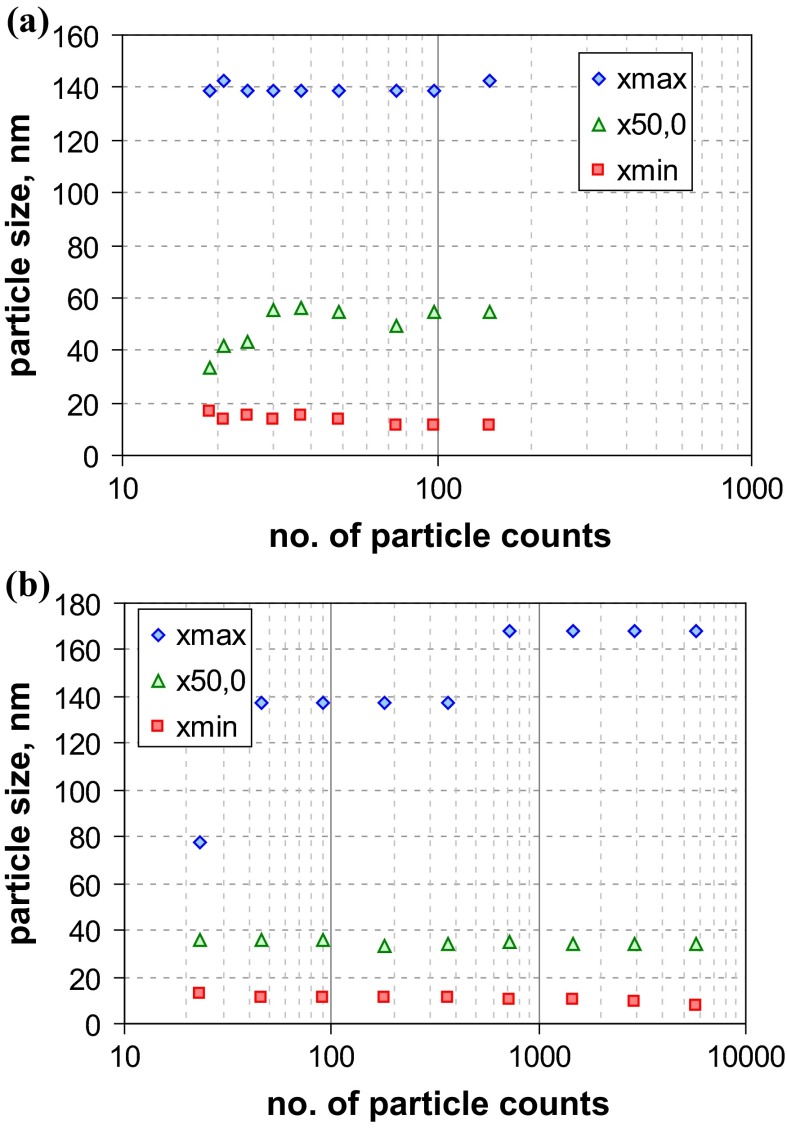
Impact of no. of counted particles on the parameters of number-weighted size distributions by SEM (maximum, median and minimum size); for **a** QCM6 and **b** RTM1

Examples of results for QCM6 and RTM1 are plotted in Figs. 8 and 9; they reveal that the size distributions converge, as the number of examined particles is increased. In particular, the shape of the distribution function becomes smoother and more accurately resolved at its edges. However, the median values *x*_50,0_ prove to be relatively robust parameters. For the materials of this study as few as 200 particles were sufficient to keep the deviation from the largest sample below 2 %. This comes in agreement with the results of de Temmerman et al. (2014a). The relatively high robustness of the *x*_50,0_ results from the fact that relevant stochastic fluctuations concern only two size classes (*x* ≤ *x*_50,0_ and *x* > *x*_50,0_), which both comprise a relatively high number of particles. Finally, we emphasise that an accurate determination of *x*_50,0_ requires that the particles available for the image analysis constitute a representative sample of the disperse system.

### Influence of the characterisation methodology on the quality of measurement data

As explained in the experimental section, this study ensured defined conditions for the whole analytical chain by providing protocols, guidelines, and data sets to all participants. However, what is a prerequisite for comparing different results may also introduce bias into the analytical results, for instance, when the procedures described in protocols are inappropriate for a given material or when instrument software is fed with erroneous model parameters. This section illustrates the importance to properly define the conditions of measurement.

#### Impact of sample preparation

As mentioned above, the comparison among different MTs relies on uniform sample preparation. In the context of material classification according to the NM definition, it is further necessary to achieve the highest feasible degree of desagglomeration for particle characterisation. Beside high-pressure dispersion ultrasonication has proved to be the most effective way of disintegrating agglomerates and aggregates of nanoparticles (Bałdyga et al. 2009; Pohl et al. 2005), for which reason, it has been employed as the standard dispersion procedure in this study. Dispersion protocols were optimised with regard to the acoustic energy input per suspension volume. In addition, the protocols defined appropriate dispersing agents and concentrations.

**Fig. 10:**
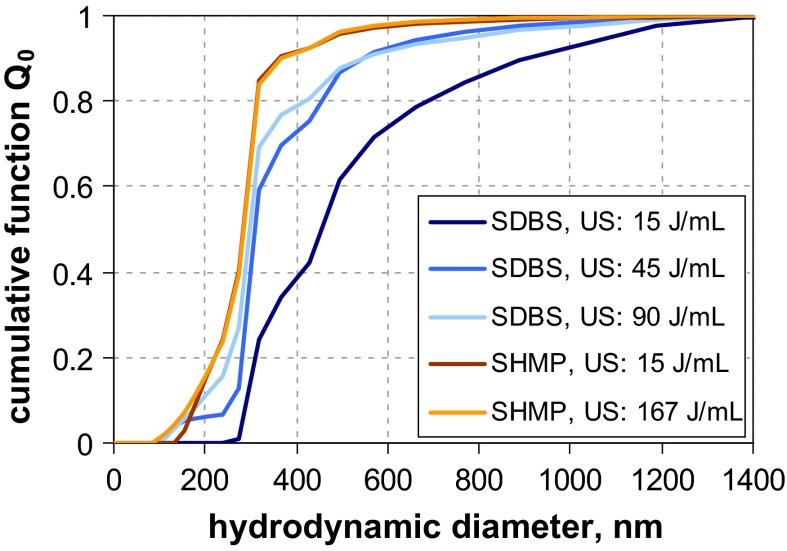
Number-weighted sum functions of RTM2 from measurements with DLS when dispersed in SDBS and SHMP for different ultrasonication energy input

Figure 10 shows the impact of both factors, dispersing agent and sonication energy, for RTM2 (BaSO4, fine grade). Two different dispersing agents were tested during the development of sample preparation protocols: sodium dodecylbenzenesulphonate (SDBS) and sodium hexametaphosphate (SHMP). For both, the acoustic energy input was varied within a range from 15 to 170 J/mL. The progress of dispersion was monitored by means of DLS; the figure shows thus derived *Q*_0_ for characteristic points of the dispersion process. Obviously, the dispersing agent is the decisive parameter of sample preparation for this case. In the presence of SHMP, brief ultrasonication suffices to virtually attain the “final state of dispersion”, SDBS is much less effective: even for relatively high sonication energy, one observes a larger fraction of coarse particles than for SHMP.

The quality of dispersion protocols is primarily important for those MTs, which rely on a mobility-related particle property (e. g., AC and DLS) or on particle volume or mass (e. g., spICP-MS). However, inadequate sample preparation can also severely affect the results of imaging techniques (e. g., SEM). Consequently, individual dispersion protocols were developed for each RTM and differentiated for the MTs if necessary. They are publicly available (cf. supplementary material).

#### Impact of data analysis

Data analysis is a further critical issue within the analytical chain. However, while inappropriate sample preparation frequently leads to visual effects of the suspension produced (e. g., flocculation), artefacts due to wrong data analysis usually remain concealed. Two aspects of data analysis are considered here: the dependency on model parameters and the impact of inversion algorithms; both are also related to the conversion of intrinsically measured size distributions into number-weighted ones.

**Fig. 11:**
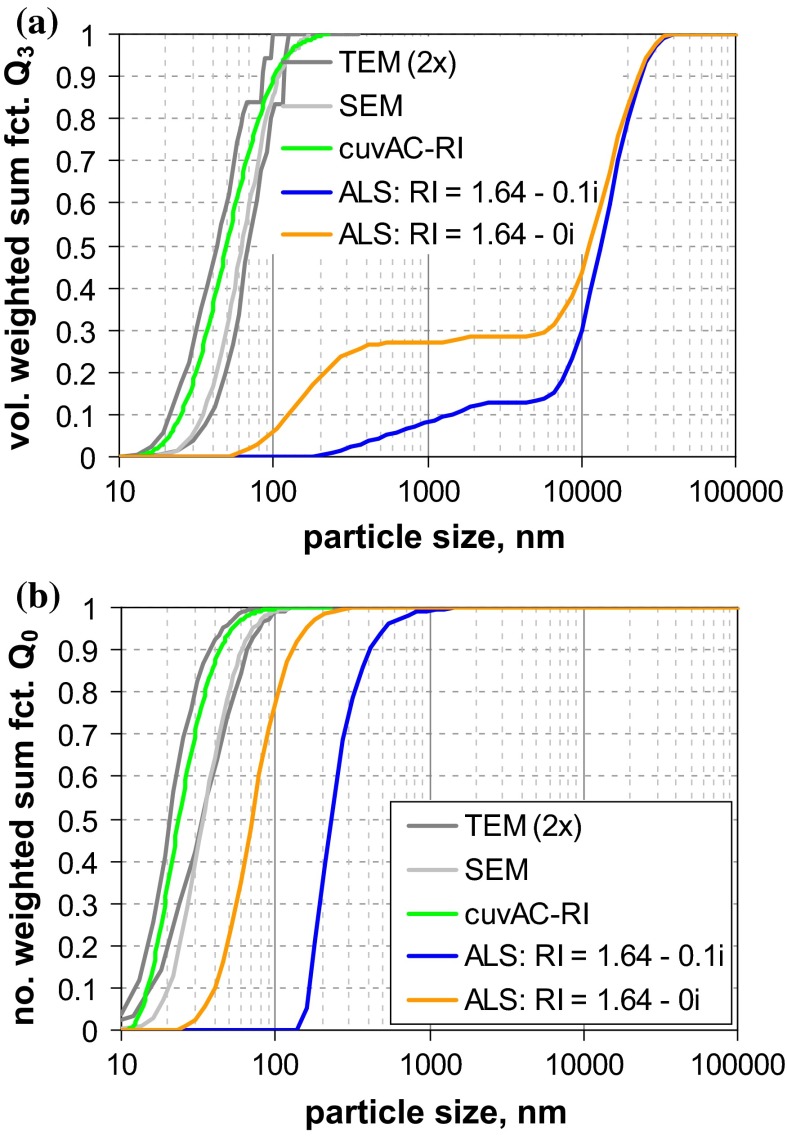
ALS result for RTM1, impact of RI values on **a** volume-weighted size distribution and **b** number-weighted size distribution (additionally results of TEM (2×), SEM and cuvAC-RI)

Apart from imaging techniques, all MTs rely on models that relate the measured signals to particle size and also to particle number. These models often require values of certain material properties as input parameters (cf. Table S-2, supplementary material S.4). Optical MTs normally need the particles’ refractive index (RI) either for sizing (e. g., ALS) or for conversion to *Q*_0_ (e. g., AC-turb). Figure 11 shows an example for the influence of RI on the result of the ALS analysis. The graphs refer to representative test material RTM1 and display measurement results of TEM, SEM, and cuvAC-RI (i. e., MTs whose resulting size distribution is independent of a specific RI value; note that cuvAC-RI measures the RI of the sample but does not require the particles’ RI for data evaluation) as well as those of ALS when analysed with a real part only and with a complex value of the particle refractive index. Adding an imaginary part of 0.1 i to the RI (which is actually real) yields a significant change in *Q*_3_ (Fig. 11a) and a large shift in *Q*_0_ (Fig. 11b). The comparison with AC confirms that the correct RI value yields plausible results for *Q*_0_.

A further example of the potential impact of RI on ALS results is shown in Fig. S-20 for RTM4, which is CaCO_3_ powder in its calcite phase. Calcite consists of elongated crystals, for which the RI depends on the crystal orientation. There are two principal RI values: for the “ordinary ray”, i. e., axis is parallel to incident light, and for the “extraordinary ray”, i. e., axis is parallel to polarisation of incident light. The corresponding values are 1.66 and 1.48, respectively. In practice, crystals will be randomly aligned during an ALS measurement zone, thus the orientation averaged RI (1.53) applies. The example demonstrates that data analysis with the faulty RI value can result in huge mis-evaluation of the number-weighted median. Again, the impact of erroneous RI value is considerably amplified at conversion.

**Fig. 12:**
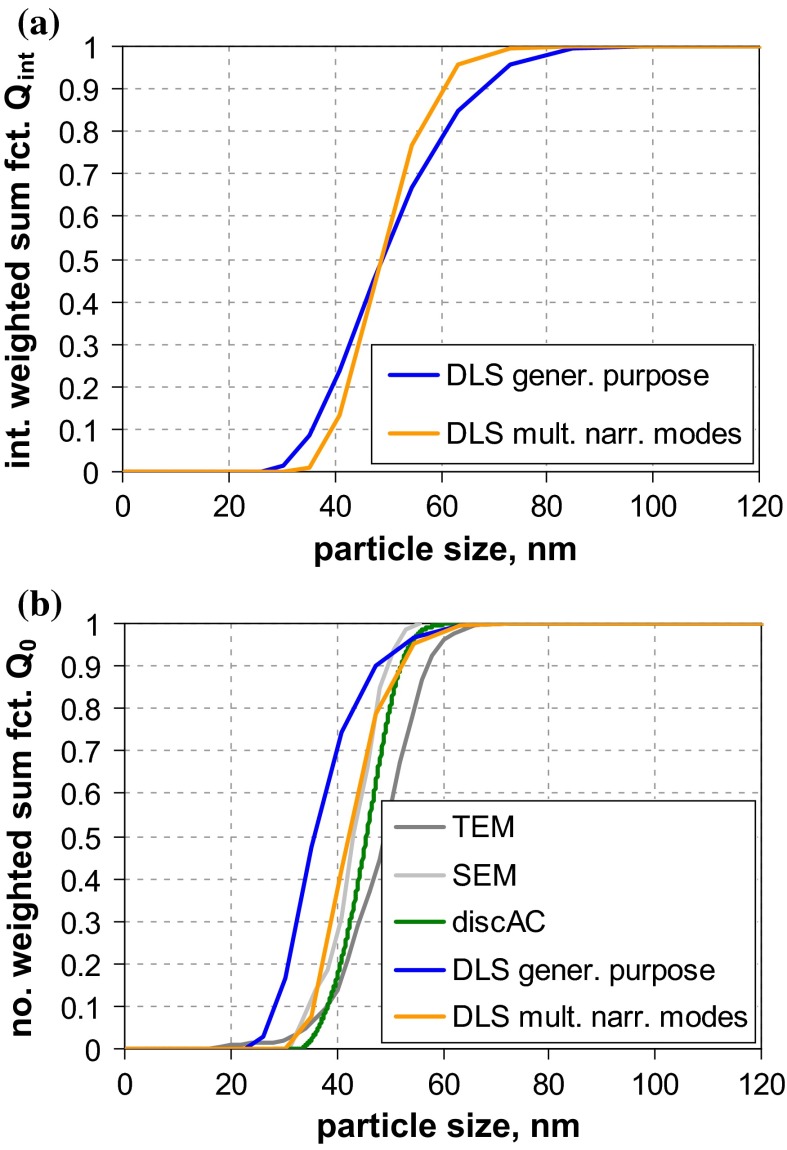
DLS result for QCM1, impact of analysis settings (“general purpose”, “multiple narrow modes”) on **a** intrinsically measured intensity-weighted and on **b** derived number-weighted size distribution (in case of the latter: additionally results by TEM, SEM, and discAC-turb)

Spectroscopic MTs, such as DLS, ALS, SAXS, and USSP, derive the size distribution from a distributed signal (spectrum), for which purpose numerical algorithms are employed that impose bias on the distribution shape (e. g., on smoothness and non-negativity). The outcome of this spectrum inversion depends on the selected algorithm and its parameterisation. This is shown in Fig. 12 for the analysis of DLS data of QCM1. With respect to the intrinsically measured *Q*_int_, the algorithm settings only affect the distribution width, but not the median size. However, after converting into *Q*_0,_ one observes a significant impact of the algorithm settings on the number weighted median (cf. Fig. 12b).

**Fig. 13:**
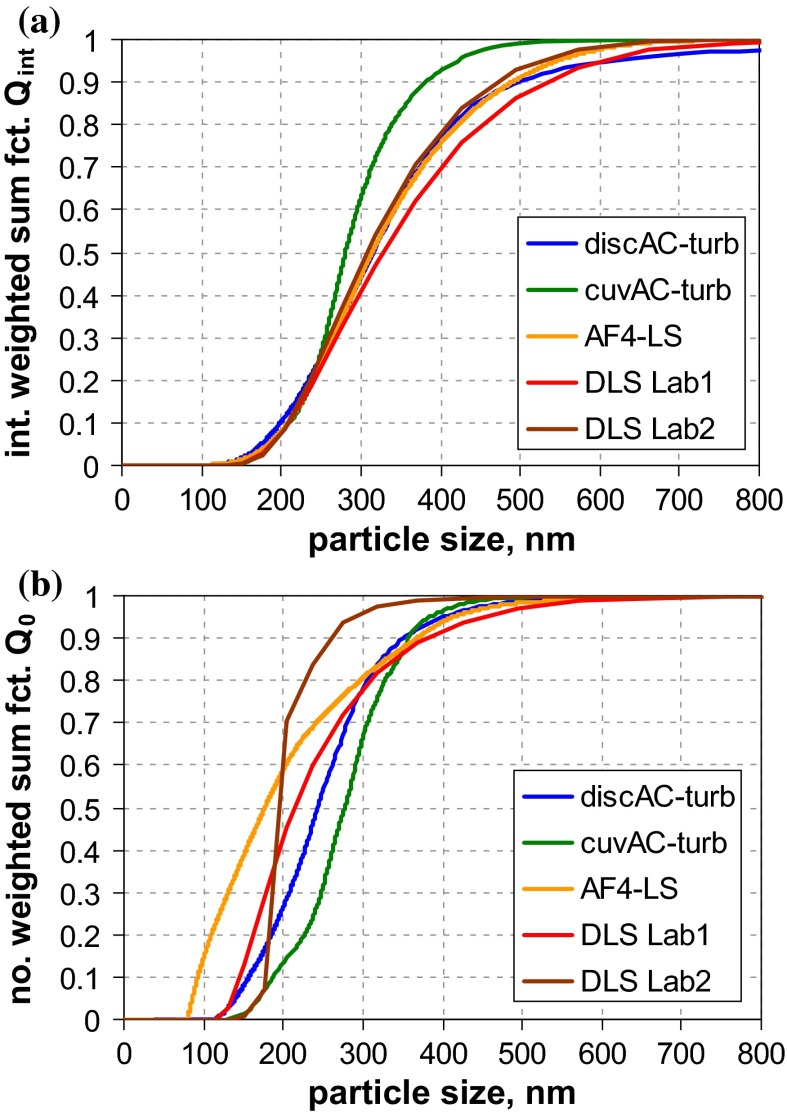
RTM3 (coated TiO_2_) results of mobility-based MTs with particle quantification by light extinction or light scattering; **a** intrinsically measured intensity and extinction-weighted and **b** derived number-weighted sum functions

All three examples on data analysis demonstrated that for non-counting MTs, the detrimental effect of inappropriate data analysis may become significantly magnified by conversion. This effect applies to any perturbation of the measured size distribution, e. g., caused by improper dispersion procedures, agglomeration or contaminant particles. A kind of worst-case scenario is shown in Fig. 13, which plots the intrinsically measured size distributions (*Q*_int_ or *Q*_ext_) and *Q*_0_ of RTM3 for AC techniques, AF4-LS and DLS (all are mobility-based). The results are rather similar when presented as *Q*_int_ or *Q*_ext_ (cf. Fig. 13a), whereas conversion into *Q*_0_ leads to considerable differences (Fig. 13b).

**Fig. 14:**
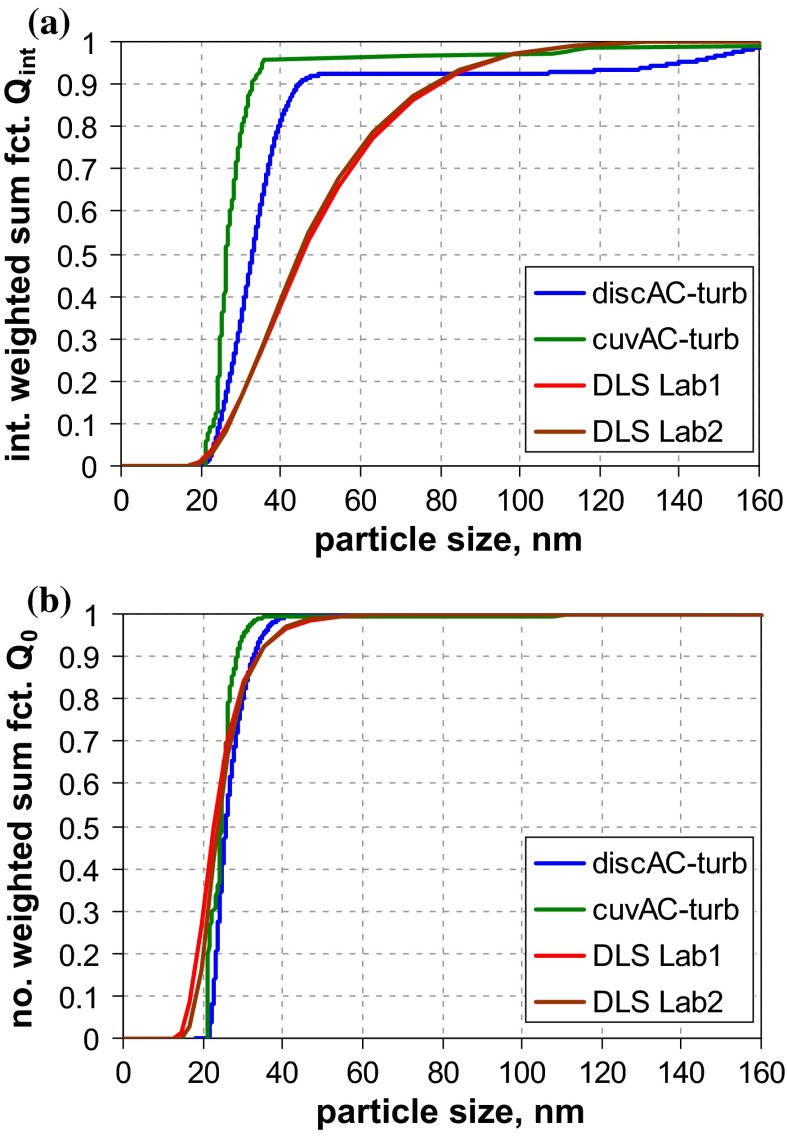
QCM2 (nano SiO_2_) results of mobility-based MTs with particle quantification by light extinction or light scattering; **a** intrinsically measured intensity or extinction-weighted and **b** derived number-weighted sum functions

However, conversion may also give rise to a suppression of differences that occur in *Q*_int_ and *Q*_ext_. Such a “harmonisation-scenario” was encountered for QCM2 (Fig. 14), where clear differences among the intrinsic results are seen. The origin of this effect is not definitely clear, but is certainly related to a different sensitivity towards coarse particles/agglomerates or due to loss of sample stability in some measurements. After conversion to *Q*_0,_ the coarse particle fractions virtually disappeared. Similar observations were made for RTM1 (cf. Fig. S22).

#### Impact of data pre-treatment

**Fig. 15:**
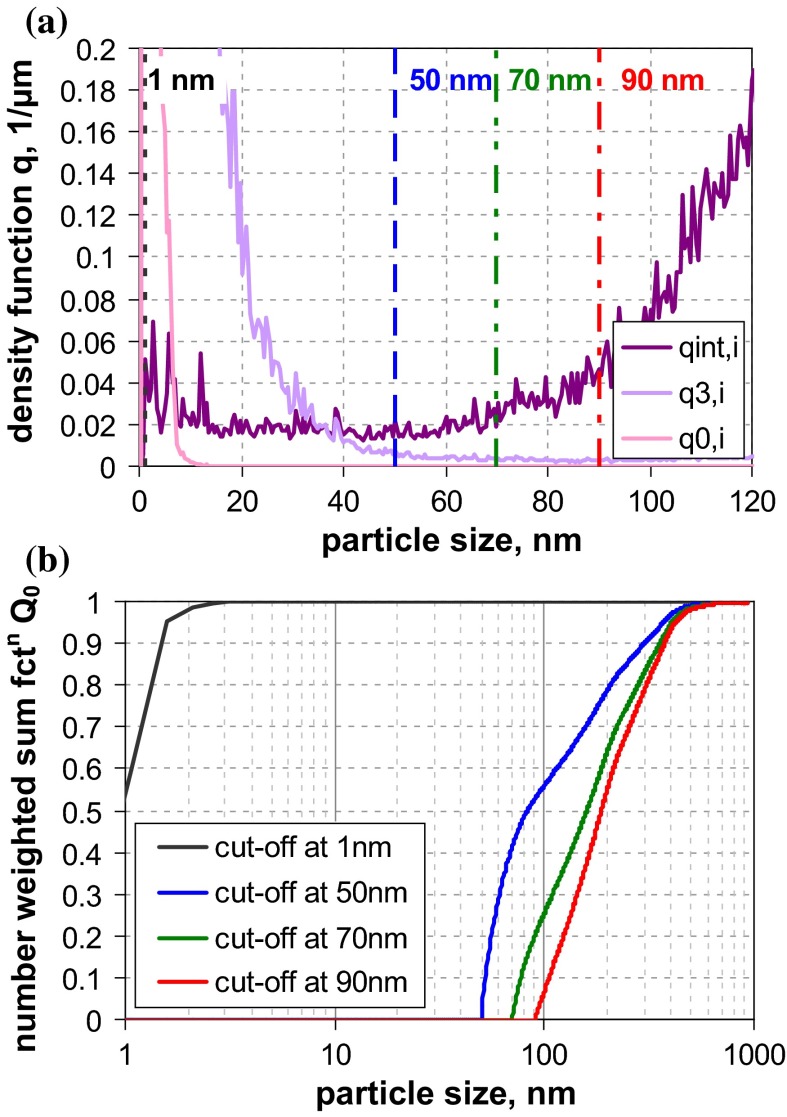
Impact of setting a lower size limit on *Q*_0_ for RTM3 (coated TiO_2_) when measured with AF4-LS; **a** detail of density functions for unbound size range and indication of possible cut-off values for the lower size limit, **b** sum functions of number-weighted size distribution for different values of lower size limit

A further aspect of the reliability of measured size distribution is the restriction of the *Q*_3_ size range and its implications on the conversion to *Q*_0_. The size range spanned by the measured size distribution is primarily determined by the sensitivity of the MT (e. g., to scattered light) but can also be defined by the settings of the analysis algorithm. Specifically, for spray-DEMA, discAC-turb, and AF4, the lower size limit has to be selected or confirmed by the operator, to remove “residual” particles, handle baseline shift, and eliminate the void peak, respectively (cf. supplementary material S.2). This setting of the lower size limit may severely affect the NM classification, as demonstrated in Fig. S23 on results for discAC-turb and in Fig. 15 on results for AF4-LS: the wider the size interval is chosen, the stronger the 1/size^3^ factor during conversion suppresses those modes that are statistically significant in *Q*_3_. Effectively, the conversion to *Q*_0_ amplifies noise and delivers completely misleading results. Consequently, within this study, any data treatment is considered to have followed defined and harmonised rules to cut off “residual particles” if the local minimum in the fractional number concentration was clearly separated from the main size distribution.

### Consequences for the implementation of the recommended NM definition in industrial practice

Our study on the characterisation of QCM particle systems and commercial powders with the most common MTs for particle sizing has clearly demonstrated that there is no single MT that ensures reliable identification of NM for all kinds of materials. This also applies to imaging techniques, such as TEM or SEM, which are frequently considered as the only possibility to finally suggest whether a material is an NM. Since the expenditure of time and staff for imaging techniques is rather high, there is a strong demand for MTs allowing a fast, cheap, and reliable classification of materials that are definitely nano or clearly non-nano. This leads to a tiered characterisation strategy.

#### Tiered approach (for high-reliability NM classification)

The proposed approach distinguishes three levels of characterisation methods or MTs. *Tier* *1*—*powder* facilitates a screening based on integral properties of powders. In contrast, *tier* *1*—*suspension* aims at the determination of size distributions of suspended, i. e., individualised, particles. However, the measured distribution functions are not intrinsically number-weighted or not related to the constituent particles (but to the morphology of aggregates). In spite of this, these MTs can be used for a screening decision. While the analysis within any of the *Tier* *1* MTs may be fast, it does not warrant high certainty with respect to NM classification. Unambiguous decisions are only possible within *Tier* *2* (confirmatory techniques), in which *Q*_0_ of (constituent) particles are directly measured and their median values (*x*_50,0_) can be thus determined with high accuracy. More details on an integrated decision tree are prepared for publication in the NanoDefine Technical Report D7.10 (www.nanodefine.eu/index.php/downloads/nanodefine-technical-reports).

The analytical strategy of a tiered approach is to quickly conclude on the type of a material within *tier* *1*. On this level the critical quantity, i. e., *x*_50,0_, is only indirectly measured or derived from an empirical correlation rule, which means that any prior knowledge on the material (e. g., by qualitative imaging) should be used to support the decision if nano or non-nano. In the best case, *tier* *1* leads to a clear statement that a material is nano or not; otherwise, confirmative techniques from *tier* *2* have to be employed.

Good candidates for *tier* *2* are evidently EM techniques. The measurement results of *TEM* and *SEM* (even among different instruments) were within a factor 1.2 for half of the materials, within a factor 1.5 for many, and a factor 2 in the worst case, resulting in a consistent NM classification for all tested QCMs and RTMs. In addition, they completely cover the relevant size range. On the other hand, our results indicated that sample preparation is a crucial issue for the reliability of EM results—in particular, for highly polydisperse materials (cf. Fig. 2b)—and that for platelet-like particles, the smallest external dimension is difficult to assess from two-dimensional images. Other authors (Jung et al. 2002) reported on the shrinkage of particles during the TEM analyses (due to vacuum and electron beam), which can further affect the classification of materials. Hence, EM techniques will serve as a powerful tool for the NM classification, yet their applicability does not cover all materials. In general, the plausibility of EM results should always be checked (e. g., with BET) before being used for classification.

The most prominent example for a *tier* *1*—*powder* technique is the determination of the VSSA by means of gas adsorption measurements according to the *BET* method. This selection supposes that the VSSA or the BET equivalent minimum size *x*_BET,min_ (cf. Appendix 7) strongly correlates with the number weighted median *x*_50,0_ of the smallest external dimension of the (constituent) particles. This is obviously difficult to be ensured for all types of materials, but for the materials of the present study, *x*_BET,min_ deviated from *x*_50,0_ by SEM or TEM within a factor 1.5 for half of the materials, within a factor 2 for many, and a factor 2.5 in the worst case, and is hence good enough for screening down to a borderline region. This finding is rationalised by the BET metrics depending on the minimum size of constituent particles without the need to disperse and disaggregate. However, BET fundamentally cannot provide size distributions. Employing BET within a tiered approach for NM classification would be highly attractive, since BET results are frequently employed by industry to distinguish different grades of particulate materials.

For *tier* *1*—*suspension*, there are quite a lot of potential MTs, but not all of them provide the required applicability to size range and not all reliably determine *Q*_0_. In view of the results of our analytical study, we propose *spray-DEMA*, all *AC* techniques, and *DLS* as candidates for the *tier* *1*— *suspension* techniques. Similar to BET, we found for these techniques that the number-weighted median (*x*_50,0_) differed from the corresponding SEM value by a factor 2 for most materials, and a factor 2.5 in the worst cases, with only two exceptions far away from the borderline region and hence without compromise on the correctness of NM classification. Screening by *tier* *1*—*suspensions* fails for materials that are composed of highly-porous, fractal-like aggregates—e. g., pyrogenic metal oxides. Such materials should be screened within *tier* *1*—*powder*, i. e., by BET. Likewise, BET analysis is misleading for NM classification when the material is microporous (not in this study). In this case, screening should be accomplished by *tier* *1*—*suspension* techniques. These examples demonstrate the importance of the above mentioned “prior knowledge” for the selection of a characterisation strategy and the interpretation of its results.

#### MT candidates with potential of reliable NM classification

Our recommendations reflect the current state of the art and the results on real-world materials as obtained in the present study. Yet, there are more promising candidates within the present study, which experience on-going developments driven by the need for the accurate characterisation of NMs.

For instance, the element-selective detection principle of *spICP-MS* results in a restriction of applicability that is inherent to the technique, but it also represents a unique asset for the selective analysis of particles contained in formulations and consumer products; improvements in the sensitivity for lighter elements have been achieved, and also the lower detection limit of particle size is constantly improving. The size range limitation is removed by the optional coupling of *AF4-ICP-MS*, which bears equally great potential especially for complex formulations, but was not ready for evaluation in the current study. The well-established *AF4-LS* was limited by the LS detection principle in some cases, but the final conclusions cannot be drawn yet, due to the lack of data.

The applicability of *PTA* to the nano-range is currently restricted to a small range just below 100 nm or to strongly scattering materials. To consider PTA as a screening technique, it is necessary to enlarge the accessible size range to smaller values. This required enhancement of sensitivity may be realistically achieved within the next years.

A further potentially interesting technique is *SAXS*, which has proven as an excellent analytical tool for the characterisation of nanoparticles in this study. However, it obviously fails to correctly classify non-nano materials. It would be useful to expand the upper limit from approximately 100 nm to the µm-range, which needs SAXS instrumentation for highly intense X-rays scattered at ultra-small angles (USAXS)—currently only available at synchrotrons. Whether this can be realised in laboratory instrumentation is questionable. An alternative approach might be to employ SAXS for the measurement of the specific surface area of the (constituent) particles.

Currently, *ALS* is also excluded from the tiered approach, because results in the submicrometre range are not very consistent (in contrast to those for *x* > 1 µm). The reasons are rather fundamental (light scattering pattern of nanoparticles that are not finely structured and are not very intense). Additional problems may result from the aspiration of commercial instruments to measure nanoparticles and micrometre particle with one optical setup, restricting to particles below 1 µm could improve the situation, but is not very likely to be seen in commercial instruments.

Finally, the analytical study cannot currently encourage to use *USSP* within *tier* *1* because of the practical limitations observed. Even though the MT exists in different types of instrumental configurations (sample size from a few µL to hundreds of mL), its major restriction is the need for particle concentrations in the order of vol%. Moreover, the conversion into *Q*_0_ sometimes yielded results beyond any physical meaning, which indicates that data analysis should be still improved.

## Conclusions

The implementation of the EC recommendation for a definition of nanomaterial (NM) in industry and legal institutions is a tremendous analytical challenge. We evaluated the performance of MTs on both quality control materials (QCMs) and on real-world particulate materials, and found that no single MT can be recommended for guidance. Required is a tiered approach that combines different MTs and employs prior knowledge on the material (physico-chemical properties, including general morphological properties of the particles). The tiered approach comprises screening techniques (*tier* *1*—*powder* and *tier* *1*—*suspension*) as well as confirmatory techniques (*tier* *2*—*imaging*). *Tier* *1* techniques are intended to provide clear statements whether a material is an NM, or whether more profound analyses by *tier* *2* techniques are required, because the number-weighted median *x*_50,0_ is close to the borderline of 100 nm. They either probe integral properties of the particle system (e. g., VSSA) or determine the distribution of equivalent diameters rather than the geometric lengths of the external dimension. In addition, the intrinsically measured size distributions are typically non-number-weighted. For this reason, the *tier* *1* techniques are expected to perform well for low and moderate polydispersity. Moreover, most techniques that are relevant for *tier* *1*—*suspension* cannot resolve the internal structure of particles aggregates, instead they probe aggregate properties. Their ability to reliably assess particulate materials according to the EC definition is, therefore, restricted to materials that consist of particles as individual entities or of well-dispersible aggregates. Based on our data, matching nano/non-nano classification by both *tier* *1*—*suspension* and *tier* *1*—*powder* indicates that this validity criterion is fulfilled. Otherwise, *tier* *2*—*imaging)* can help, but even then ambiguity remains.

This study yields recommendations for the MTs that can be attributed to the different tiers based on their proven performance for real-world materials.

*Tier* *1*—*powders* can rely on BET, but only outside the borderline region, whose limits we explore in details elsewhere (Wohlleben et al., Reliable nanomaterial classification of powders using the volume-specific surface area method; submitted to *Nanoscale*).

*Tier* *1*—*suspension* can be realised with spray-DEMA, all AC techniques or DLS, yet similar as for BET, they are inconclusive for a borderline region and certain particle morphologies. Further candidates for *tier* *1*, which we tested, provided no reliable classification (ALS, PTA, and SAXS), or were not ready for a final assessment (AF4 and spICP-MS).

*Tier* *2*—*imaging* measurements can be conducted with TEM or SEM, which give access to the constituent particles of aggregates and to the smallest external dimension of particles for most materials. However, the preparation of representative samples constitutes a major source of uncertainty and ambiguity for *tier* *2*, and the determination of the smallest external dimension remains challenging (if possible at all) for several classes of morphology, e. g., for three-dimensional aggregates and two-dimensional platelets.

Inconsistent results occur with both *tier* *1* and *tier* *2* techniques for highly polydisperse samples: for most screening techniques because of their relative insensitivity towards the fine size fractions and for imaging techniques, because any particle deposition process on substrates is affected by particle size. We also observed ambiguity in *tier* *1* and *tier* *2* results when the materials were composed of indispersible aggregates comprising a large number of constituent particles. In these cases, the *tier* *1* – *powder* might be preferable for a pragmatic implementation. Despite these challenges, our results suggest that reliable NM identification is possible for a broad range of real-world substances, provided they are not borderline cases (i. e., if their *x*_50,0_ is outside the 50 to 150 nm range). In this size range, conflicting results are to be expected also from EM labs, and weight of evidence approaches might be required to combine evidence from all tiers.

Finally, we can extrapolate from our study that the classification of mixtures of different substances is probably rather difficult and prone to artefacts. The interpretation of particle sizing results for such materials can be critically misleading if, for instance, the turbidity of a mixture is solely determined by one light-absorbing component (e. g., in AC-turb), or when the scattering signal of a mixture is dominated by the component with highest optical or electron density contrast (e. g., DLS and SAXS).

### Electronic supplementary material

Below is the link to the electronic supplementary material.

Supplementary material 1 (PDF 1743 kb)

## Figures and Tables

**Fig. 16:**
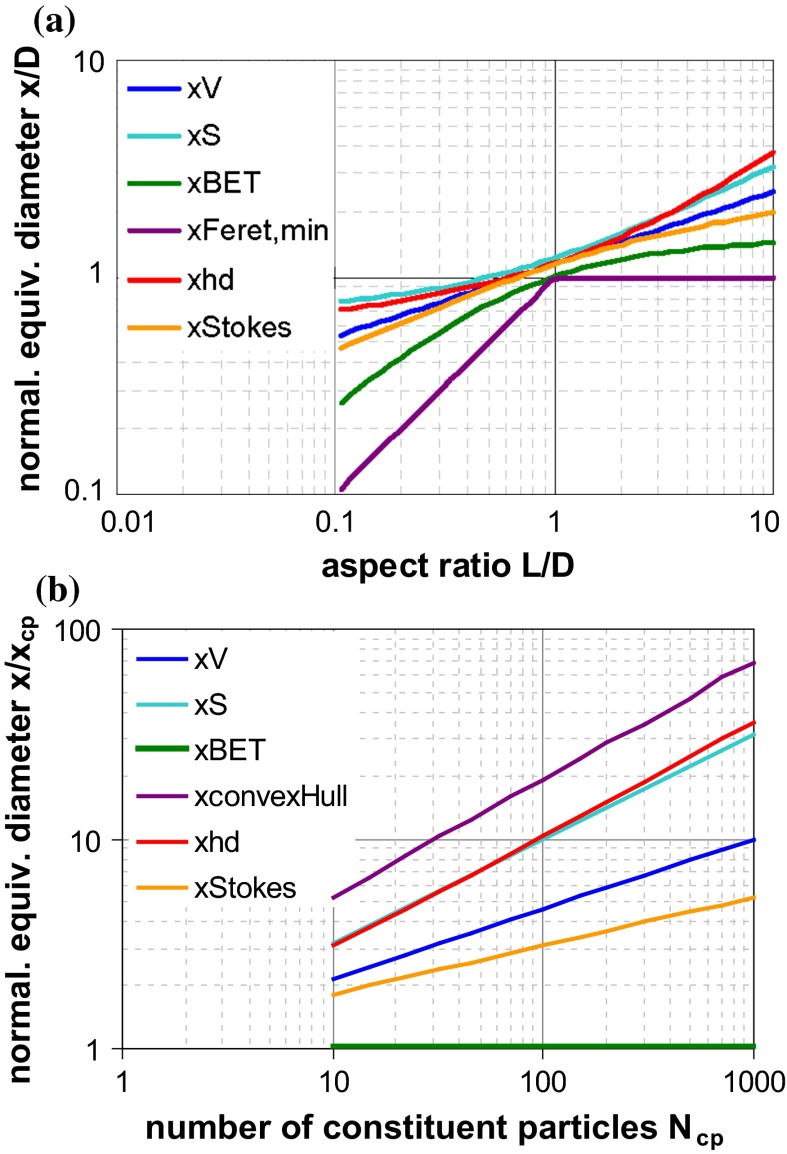
Normalised equivalent diameters of **a** cylindrical particles (diameter *D* and length *L*) and **b** DLCA-like agglomerates (spherical constituent particles of diameter *x*_cp_); equivalent diameters with respect to volume (*x*_V_), surface area (*x*_S_), specific surface area (*x*_BET_), hydrodynamic mobility (*x*_hd_), and settling velocity (*x*_Stokes_); in addition minimum Feret diameter (*x*_Feret,min_) and diameter of the circumscribing sphere (*x*_convexHull_)

## Appendix 1: Brief description of materials

See Table **1**.

## Appendix 2: Sample preparation

See Table 2.

**Table 2:** Main features of the sample preparation procedures employed

Code (material)	Wetting agent	Dispersing agent	Conc. at dispersion	Volume (mL)	Pre-treatment	Energy density (kJ/mL)
RTM1 (BaSO_4_, UF)	Not required	2 mg/mL SHMP	0.1–0.26 wt%	2…100	Vortexing	0.2…5
RTM2 (BaSO_4_, fine)	Not required	2 mg/mL SHMP	0.1–0.26 wt%	2…200	Vortexing	0.3…5
RTM3 (coated TiO_2_)	Ethanol	2 mg/mL SHMP	0.01 wt%	2…100	US bath	0.3…5
RTM4 (CaCO_3_)	Not required	2 mg/mL SHMP	5 wt%	2…70	Stirring	0.3…2
RTM5 (kaolin)	Not required	0.1 mg/mL TSPP	6 wt%	2…70	US bath	0.3…3
RTM6 (fumed SiO_2_)	This material was provided as suspension					
RTM7 (pigment Y83)	Methanol/2-propanol	0.5 wt% SBNS	0.01–0.1 wt%	2…100	Complex	0.3…5
RTM8 (pigment Y83)	Methanol/2-propanol	0.5 wt% SBNS	0.01 wt%	2…100	US bath	0.3…1
RTM9 (BMC)	Stearic acid	SDS	1 wt%	2…100	Stirring	0.3

Dispersing agents:

*SDS* sodium dodecyl sulphate (CAS No. 151-21-3)

*SHMP* sodium hexametaphosphate (CAS No. 10124-56-8)

*SBNS* Sodium butyl naphthalene sulphonate (CAS No. 52628-07-6)

*TSPP* Tetra-sodium pyrophosphate (CAS No. 7722-88-5)

## Appendix 3: Brief description of the employed measurement techniques

### Transmission electron microscopy (TEM) and scanning electron microscopy (SEM)

These analytical techniques rely on the various interactions of accelerated electrons with a specimen (Flegler et al. 1993). Eventually scattered or transmitted electrons are used to create highly resolved images of the specimen (i.e., down to a few nm or even below). The distinction between TEM and SEM refers to the different detection modes and coincides with different electron energies, thus maximum attainable resolution. This classical scheme is currently becoming obsolete, since hybrid techniques (e. g., SEM in transmission mode—TSEM) steadily gain attraction (Klein et al. 2011; Rades et al. 2014). In the context of particle characterisation, TEM and SEM are highly appreciated for visualising the particles’ morphology (shape, state of aggregation, etc.). Yet, they can also reliably be employed for the determination of particle size distributions (Meli et al. 2012). The key issue is the preparation of a representative sample—in particular, for high polydispersity, considerable variation of particle shape or mixture of different particulate phases. In addition, instrument specific effects, such as the high sensitivity of SEM InLens detectors to surface charging, may affect the accuracy of size measurement (Hodoroaba et al. 2014).

### Single particle inductively coupled plasma mass spectrometry (spICP-MS)

Mass spectrometers operated in a mode of high time resolution are able to distinguish between the background signal of dissolved matter and peak signals originating from isolated particles. In this way, it is possible to measure the mass of individual particles (Peters et al. 2014). The minimum size that can be resolved depends on the chemical composition of the particles, since the intensity of ICP-MS signals correlates with the atomic weight of the elements. The nano-range is accessible for most metals and the corresponding metal oxides. However, this also means that spICP-MS does not support the NM classification for a significant number of particulate materials, including organic pigments and silica products.

### Particle tracking analysis (PTA)

The technique is based on the visualisation of fine colloidal particles by their scattered light when illuminated against a dark background (dark field microscopy and ultramicroscopy). The inherent Brownian motion of such fine particles leads to a steady displacement of the observed scattering pattern, which can be quantified as average displacement length per time step and thus reveals the translational diffusion coefficient of each particle (Saveyn et al. 2010). Though commercial instruments are rather new, the technique was already demonstrated by Perrin (1909) for particles in 1909.

### Differential electrical mobility analysis on sprayed suspensions (spray-DEMA)

DEMA is a classical technique for aerosol characterisation, which consists of three components: defined charging of the particles, their classification according to the electric mobility, and the quantification of classified particles. The most common system, also employed in this study, is based on a bipolar charger, a sequential aerosol classification process and an optical condensation particle counter. It allows the determination of fractional number concentrations for particles in the size range of 10 nm to approximately 1 µm (Fissan et al. 1996; Motzkus et al. 2013). For non-spherical particles, the MT probes the *mobility equivalent diameter*, which is very similar, but not identical to the hydrodynamic equivalent diameters measured with PTA, AF4, or DLS (i. a., because of the velocity slip at particle surface). In this study, DEMA was solely applied to aerosolised particles from (aqueous) suspensions. The aerosolisation was achieved by conventional atomisation (purely mechanical spray generation) and electro-spray generation (spray generation in an electric field). For practical reasons, the latter was only applied to materials with maximum particle size below 200 nm.

### Analytical centrifugation (AC)

Centrifugation results in a classification of suspended particles in accordance to their mass and hydrodynamic mobility. Both properties define the settling velocity or more generally the sedimentation coefficient; the corresponding equivalent diameter is called Stokes diameter. There are different technical realisations of analytical centrifugation. Disc centrifuges inherently measure a scaled density function of the size distribution, whereas cuvette centrifuge primarily determine the cumulative function. Further differences are related to the quantification of particles. This study employs turbidity detectors and interference optics. The former weigh size fractions with respect to the particle extinction cross section, the latter by means of the relative refractive index increment. That means that for instance, for non-opaque nano-particles, the measured size distribution is intrinsically x^6^-weighted when using turbidity sensors, while refractive index measurements intrinsically yield an x^3^-weighted size distribution.

### Asymmetric flow field-flow-fractionation with light scattering detection system (AF4-LS)

This MT is a field-flow-fractionation technique, for which the cross field is a volume flux through one wall of the fractionation channel (von der Kammer et al. 2005). This has the advantage that both, migration to wall and counteracting diffusion, are both determined by the particles hydrodynamic mobility. In addition, this principle facilitates classification down to 1 nm. The eluent of the fractionation channel contains (more or less) narrow size fractions, the mean size of which increases in a defined manner with retention time (normal mode of operation). The concentration of each size fraction is measured by an appropriate detector. In this study, a light scattering detector was used for this purpose.

### Dynamic light scattering (DLS)

The Brownian motion of colloidal particles results in an erratic fluctuation of scattered light, which in turn can be used to determine the diffusion coefficient of the particles or the distribution of the hydrodynamic (equivalent) diameter. There are different technical realisations of DLS, which differ with respect to the impact of scattered light and to the quantification of the intensity fluctuation. Yet, all of them intrinsically measure a (scattered light) intensity-weighted size distribution (Lamberty et al. 2011; Xu 2000).

### Angular light scattering (ALS)

This term comprises a set of MTs that were originally developed for different size ranges and thus deviate considerably with respect to technical design and performance in particle sizing (Xu 2000). All instruments employed in this study are designed for measuring particles up to several hundreds or thousands of micrometres, for which reason they highly resolve the scattering pattern at small scattering angles (traditionally called *laser diffraction spectroscopy*). In addition, they measure the scattering intensity at moderate scattering angles and even in the backward direction (traditionally called *static light scattering*). One instrument also uses a wavelength shift in order to increase the sensitivity for particles below 100 nm. It should be noted that previous interlaboratory studies evaluated the performance of such hybrid ALS instruments as rather weak for the submicrometre range (Kuchenbecker et al. 2012; Mori et al. 2007). The dependence of the light scattering pattern on the particle morphology is not simple and depends on size range, principal optical properties and particle alignment. For large micrometre particles it essentially reflects the orientation averaged projection area, while for nanoparticles it depends on the orientation averaged pair distribution function of surface elements.

## Small angle X-ray scattering (SAXS)

The small wavelengths of X-rays allow a characterisation of structures and objects in the nano-range and even sub-nano range (Glatter and Kratky 1982; Meli et al. 2012). With regard to particle size analysis, only small scattering angles are relevant. In contrast to numerous other MTs, SAXS facilitates the distinction between aggregates and constituent particles. Moreover, it can also roughly resolve the shape of the constituent particles, which is important in context of the recommended NM definition. When SAXS is employed for the determination of size distributions, it relies on scattering signals, which are weighted by the particle surface. However, in practice, the calculated size distribution is either presented volume or number-weighed—a procedure that is also applied for ALS.

## Ultrasonic attenuation spectroscopy (USSP)

The manifold interactions between particles and acoustic fields result in sound attenuation and a shift of the sound speed. For colloidal particles, the interactions are mainly dissipative (i. e., sound scattering is negligible) and are related to the visco-inertial and thermal coupling between particles and continuous phase. The relevance of these phenomena depends on material properties and the ratio of particle size to wavelength. Scanning through a certain frequency range thus allows for the determination of particle size (Challis et al. 2005; Dukhin et al. 2012). Size distributions measured by USSP are volume weighted (to first approximation), which ensures the applicability to a broad measurement range. The impact of shape cannot be described in simple and at once accurate and universal terms. If the deviation from spherical shape is not too large, the equivalent diameter is approximately VSSA equivalent (Babick and Richter 2006). In contrast to most other MTs, USSP requires relatively large particle concentrations (>1 vol%), since the contribution of particle to sound attenuation has to be significant against the sound absorption in the dispersion medium.

## Gas adsorption analysis based on the method of Brunauer, Emmett, and Teller (BET)

Gas adsorption on powders is a long-established way for the determination of the specific surface area. The specific BET method of measurement and data analysis is frequently used to distinguish different grades of particulate products, even though the weaknesses of this method are well known (e. g., its insensitivity to microspores and its dependency on local distribution of surface energy). Volume specific surface areas (VSSA) are integral properties of particle systems and correspond to a characteristic mean size. If the principal shape of the particles is known, it is even possible to calculate an average value for the smallest external dimension of the particles.

See Table 3.

**Table 3:** Brief description of measurement techniques (MT) employed in this study

MT	Type of MT	Particle property	Intrinsic result	Sample form	Standards
TEM	Counting (via image analysis)	Min. Feret diameter	*Q*_0_ (*x*_Feret,min_)	Powder or suspension	ISO 29301, ISO 13322-1
SEM	Counting (via image analysis)	Min. Feret diameter	*Q*_0_ (*x*_Feret,min_)	Powder or suspension	ISO/TS 24597 ISO 13322-1
spICP-MS	Counting	Mass	*Q*_0_ (*x*_V_)	Suspension	ISO TS 19590
PTA	Counting	Diffusion coeff.^a^ (mobility-based)	*Q*_0_ (*x*_hd_)	Suspension	ISO/CD 19430
Spray-DEMA	Fractionation	Electric mobility	*q**_0_ (*x*_mob_)	Suspension	ISO 15900
discAC-turb	Fractionation	Settling velocity (mobility-based)	*q*_ext_(*x*_Stokes_)	Suspension	ISO 13318, ISO 15825
cuvAC-turb	Fractionation	Settling velocity (mobility-based)	*Q*_ext_(*x*_Stokes_)	Suspension	ISO 13318, ISO 15825
cuvAC-RI	Fractionation	Settling velocity (mobility-based)	*Q*_RI_(*x*_Stokes_)	Suspension	ISO 13318, ISO 15825
AF4-LS	Fractionation	Diffusion coeff.^a^ (mobility-based)	*q**_int_(*x*_hd_)	Suspension	
DLS	Spectroscopic	Diffusion coeff.^b^ (mobility-based)	*q**_int_(*x*_hd_)	Suspension	ISO 22412, ISO 13321
ALS	Spectroscopic	Pair distribut. of (projected) surface elements	*q**_2_(*x*_ALS_)	Suspension	ISO 13330
SAXS	Spectroscopic	Pair distr. of surface elements	*q**_2_(*x*)	Suspension	ISO 17867
USSP	Spectroscopic	Acoustophoretic mobility^c^	*q**_3_(*x*_US_)	Suspension	ISO 20998
BET	Integral	Specific surface area	*x*_BET,min_^d^	Powder	ISO 9277

^a^Translational diffusion coefficient

^b^Apparent diffusion coefficient

^c^For aqueous suspensions of most solid particles in the submicrometre range

^d^Explanation of this parameter in Appendix 7

## Appendix 4: Correlations among the various equivalent diameters

The various MTs employed in this study probe different particle properties, thus different equivalent diameters, or determine the minimum Feret diameter from particle images (cf. Table 3 in Appendix 3). For this reason, we cannot expect agreement for particle size values—apart from non-aggregated spherical particles. The deviations among the various equivalent diameters depend on particle shape and may be even employed to its identification or quantification (cf. Wadell 1932). For well-defined particle morphologies, it is possible to derive quantitative relations among equivalent diameters. Figure 16 shows such correlations for cylindrical particles (with length *L* and diameter *D*) and for fractal agglomerates of spheres (DLCA type, with *N*_cp_ constituent particles of diameter *x*_cp_). The hydrodynamic properties of the rods were calculated based on the numerical results of Ortega & Garcia de la Torre (2003), whereas the computation of the agglomerate properties followed the principles described in Babick (2016).

The two examples of particle morphology reveal some relations, which generally holds true (Leschonski 1986): $x_{\text{S}} > x_{\text{V}} > x_{\text{BET}}$, $x_{\text{hd}} > x_{\text{Stokes}}$, and $x_{\text{V}} > x_{\text{Stokes}}$.

A further interesting relation applies to both examples:

$$x_{\text{hd}} \approx x_{\text{S}} ,$$

which means that the total surface contributes to the viscous drag—a situation, which is not valid for densely packed agglomerates.

Equivalent diameters from spectroscopic MTs may defy a well-defined, unambiguous relation with other equivalent diameters. This is because the spectra of non-spherical particles (e. g., angular distribution of a scattered radiation) may qualitatively deviate from the spectra of spherical particles. The assumption of spherical shape in data analysis, then typically yields an artificial polydispersity (e. g., ALS—Matsuyama et al. 2000, e. g., USSP—Babick and Richter 2006).

## Appendix 6: Number-weighted median sizes

Table 4 shows all measured values of the number-weighted medians *x*_50,0_ and evaluates these results based on the conformity with SEM regarding the NM classification. However, classification by SEM is not necessarily correct, as the example of the kaolin sample has indicated (cf. discussion on Fig. 6).

**Table 4:** Compliance with classification by SEM based on tabulated values of the number-weighted median size *x*_50,0_ (in nm)

Code (material)	SEM	TEM	spICP-MS	PTA	DEMA spray	disc AC-tu.	cuv AC-tu.	cuv AC-RI	AF4-LS	DLS	SAXS	ALS	USSP	BET^a^
QCM1 (polystyrene)	43	**49**	n.m.	**50**	**47** **45**	**46**	**46**	n.m.	**33**	**36** **35**	n.m.	n.m.	n.m.	n.m.
QCM2 (colloidal SiO_2_)	22	**22**	n.m.	n.m.	**32** **30**	**26**	**25**	**23**	**18**	**24** **23**	**24**	n.m.	n.m.	n.m.
QCM3 (colloidal Au)	19	**18** **20**	**26**	n.m.	**16**	**18**	**17**	n.m.	n.m.	**3**	n.m.	n.m.	n.m.	n.m.
QCM4 (colloidal Ag)	5	**5** **3**	n.m.	n.m.	n.m.	**5**	**6**	**2**	n.m.	**11**	**6**	n.m.	n.m.	n.m.
QCM5 (3-mod PSL)	48	**55**	n.m.	*153* 104	**43** **46**	**50**	**52**	**52**	**48**	**80** **80**	n.m.	**52**	n.m.	n.m.
QCM6 (3-mod SiO_2_)	55	**35**	n.m.	(*166*) 100	**43** **38**	109	102	**32**	108	109 *132*	**26**	*147*	n.m.	n.m.
RTM1 (BaSO_4_, UF)	34	**33** **21**	n.m.	(*191*)	**53**	**66**	**43**	**24**	n.m.	**76** **72**	103	**76**	(**1)**	**37**
RTM2 (BaSO_4_, fine)	212	**253** **281**	n.m.	**281**	**293**	**223**	**258**	**203**	n.m.	**285**	*54*	**635** *69*	**410**	**545**
RTM3 (coated TiO_2_)	185	**180** **185**	182	**254**	**253**	**243**	**277**	**201**	**177**	**195** **215**	*80*	**483** **237**	**315**	102
RTM4 (CaCO_3_)	154	**153** **161**	n.m.	**274**	**148**	**225**	**248**	**232**	n.m.	**294**	n.m.	**160** *68*	**415**	**260**
RTM5 (kaolin)	129	**(121)**	n.m.	**210**	**252**	n.m.	**132**	98	n.m.	**290**	**187**	**152** *66*	(*1*)	*48*
RTM6 (fumed SiO_2_)	20		n.m.	(*118*) **82**	**62** **56**	**36**	n.m.	**34**	**42**	**69** **37**	**9**	**79**	**5** **12**	**14**
RTM7 (pigment Y83)	40	**39**	n.m.	*205*	n.m.	**52**	**34**	n.m.	n.m.	**68** **81**	**8**	(*1307*)	n.m.	**40**
RTM8 (pigment Y83)	157	**221**	n.m.	**(114)**	n.m.	**186**	**153**	n.m.	n.m.	**292** **269**	n.m.	**168** **133**	n.m.	**152**
RTM9 (methacrylate)	2026	n.m.	n.m.	n.m.	n.m.	n.m.	**413**	n.m.	n.m.	n.m.	n.m.	**1837**	n.m.	**4084**

*Bold* conformity with SEM evaluation, *italic* clear deviation, *underline* close to critical cut-off (100 nm), *n.m.* not measured for instrumental or practical reasons, *brackets* considered little reliable

^a^Column lists the BET equivalent minimum size, cf. Appendix 7

The table specifies the conformity with classification by SEM by means of text *font*. If the number weighted medians of an MT relates to the critical value of 100 nm in the same way as SEM does, the numbers are emboldened. A clear discrepancy is indicated by italics, whereas underlined values mean that they are close to 100 nm. This code does not represent quantitative agreement with SEM, only agreement of the resulting classification. In addition, there a few values set in brackets (e. g., USSP for RTM1), which indicates low reliability based on prior considerations (e. g., because the particle concentration was actually too low for USSP analysis). In addition, the table contains several fields filled with “n.m.”, which means that material could not be analysed due to instrumental or practical reasons (e. g., because particle size was beyond measurement range, concentration was too low, sample size too small, suspension could not stabilised for the specific MT, or foaming impeded a reliable spraying). In several instances, the table states two values, which are results by different laboratories with and without identical instrumentation (cf. Table S-1, supplementary material S.3).

## Appendix 7: BET measurements

The BET equivalent diameter *x*_BET_ is the diameter of a sphere with the same VSSA as the particle system when the VSSA is determined via gas adsorption according to the method of Brunauer–Emmett and Teller:

$$x_{\text{BET}} = \frac{6}{{S_{V} }}.$$

For long fibres or thin sheets, it is not really meaningful to work with this equivalent diameter; instead, the VSSA is used to compute the diameter of the fibre *d*_fibre_ or the thickness of the sheet *δ*_sheet_, respectively:

$$d_{\text{fibre}} = \frac{4}{{S_{V} }}$$

$$\delta_{\text{sheet}} = \frac{2}{{S_{V} }}.$$

Both parameters represent the smallest external dimensions of the considered object, which is also relevant for the NM classification. Therefore, a BET equivalent minimum size *x*_BET,min_ is employed as characteristic parameter for the NM classification (Roebben and Rauscher 2014).

$$x_{\text{BET,min}} = \frac{2 \cdot D}{{S_{V} }}$$

in which *D* indicates the number of small dimensions of the particles. The value should be set to 2 if the aspect ratio exceeds a value of 3, and to 1 if the aspect ratio is smaller than 0.25. Values of BET measurement and the corresponding *x*_BET,min_ data are listed for all RTMs in Table 5.

**Table 5:** VSSA derived from BET analyses (in mass specific surface area) and skeletal density, corresponding BET equivalent minimum size and dimensionality (cf. Table 1 in Appendix 1)

Code (material)	S_m,BET_ (m^2^/g)	ρ (g/cm^3^)	S_V_ (m^2^/cm^2^)	D	x_BET, min_ (nm)
RTM1 (BaSO_4_, UF)	36.9	4.4	162.4	3	37.0
RTM2 (BaSO_4_, fine)	2.5	4.4	11.0	3	545.5
RTM3 (coated TiO_2_)	14.8	3.99	59.1	3	101.6
RTM4 (CaCO_3_)	5.8	2.657	15.4	2	259.6
RTM5 (kaolin)	16	2.61	41.8	1	47.9
RTM6 (fumed SiO_2_)	200	2.2	440	3	13.6
RTM7 (pigment Y83)	67.7	1.484	100.5	2	39.8
RTM8 (pigment Y83)	17.5	1.5	26.3	2	152.4
RTM9 (methacrylate)	1.3	1.13	1.5	3	4084

## Appendix 8: Uncertainty with respect to repeatability (and intermediate precision)

This study aimed at a first evaluation of several MTs whether they can support the classification of particulate material according to the EC definition of NMs. For this purpose, it was necessary to cover a wide range of different materials and potential MTs, rather than to conduct an interlaboratory comparison which obeys certain metrological standards. Hence, this study gathered little data on the quality of the measured size distribution with respect to accuracy, intermediate precision and repeatability. In particular, it was not possible to quantify the “trueness” of *x*_50,0_ for most materials, as such analyses need verified reference values for a material of similar morphology. However, most participants conducted at least two repeated runs of up to three aliquots what allows assessing the uncertainty related to precision. The respective effort depended on the analysis costs (e. g., most DLS results are averaged from 30 individual measurements at 3 aliquots, while most SAXS results are averaged from just 2 individual measurements).

Corresponding values of precision-uncertainty are listed in Table 6. It is remarkable that most values are smaller than 20 %, which is clearly less than the deviation among the different MTs. For instance, the measured *x*_50,0_ values by DLS for RTM6 varied with 16 % relative standard deviation while they differ to *x*_50,0_ of discAC-turb and SEM by a factor of 1.92 and 3.5, respectively.

**Table 6:** Relative uncertainty of the number-weighted medians shown in Table 4 in Appendix 6 with respect to repeatability and intermediate precision—provided that data allowed such analysis

Code (material)	SEM	TEM	spICP-MS	PTA	DEMAspray	Disc AC-tu.	Cuv AC-tu.	Cuv AC-RI	AF4-LS	DLS	SAXS	ALS	USSP	BET
QCM1 (polystyrene)				**1.3** **%**	**0.2 %** **0.7** **%**	**4.0** **%**				5.8 %				
QCM2 (colloidal SiO_2_)				**3.5** **%**	**0.3** **%**	**3.0** **%**	16.6 %			6.3 %				
QCM3 (colloidal Au)			**4.8** **%**		**1.9** **%**	**0.1** **%**	**1.8** **%**			*32* *%*				
QCM4 (colloidal Ag)						**2.5** **%**	**1.8** **%**			7.1 %	6.2 %			
QCM5 (3-mod PSL)				**1.2 %** **1.7 %**	**1.2** **%0.7** **%**	**3.8** **%**	**3.8** **%**			9.9 %				
QCM6 (3-mod SiO_2_)				**3.4 %** **1.8 %**	**4.0** **%**	**0.6** **%**	**0.6** **%**			**3.0** **%**	**0.3** **%**			
RTM1 (BaSO_4_, UF)				6.7 %	**0.6** **%**	18.0 %	**3.9** **%**	7.0 %		**3.5** **%**				**1.1** **%**
RTM2 (BaSO_4_, fine)				5.3 %		8.0 %				**0.7** **%**				*20* *%*
RTM3 (coated TiO_2_)	**1.6** **%**		**1.6** **%**	**0.6** **%**	**0.5** **%**		**1.5** **%**			**0.6** **%**	**0.7** **%**			**2.7** **%**
RTM4 (CaCO_3_)				**1.8** **%**	**1.2** **%**					**0.4** **%**				**1.7** **%**
RTM5 (kaolin)				**1.9** **%**	**0.7** **%**		**1.9** **%**			**3.1** **%**	**0.5** **%**			**2.5** **%**
RTM6 (fumed SiO_2_)				5.4 % 7.3 %	**1.0** **%**	**2.5** **%**				16 %	**4.7** **%**		19.0 %	
RTM7 (pigment Y83)				**1.1** **%**		14 %	**1.5** **%**			9.6 %	**4.9** **%**			6.9 %
RTM8 (pigment Y83)				**1.3** **%**		**2.0** **%**	*56* *%*			0.2 %				5.1 %
RTM9 (methacrylate)														7.7 %

Emphasis indicate degree of uncertainty: *bold* for values ≤5 %, *underline* for values between 5 and 20 %, *italic* for values ≥20 %

## Abbreviations

AC: Analytical centrifugation
AF4: Asymmetric flow field-flow-fractionation
AFM: Atomic force microscopy
ALS: Angular light scattering
BET: Brunauer–Emmett–Teller method
DLS: Dynamic light scattering
DEMA: Differential electrical mobility analysis
EC: European Commission
EM: Electron microscopy
MT: Measurement technique
NM: Nanomaterial
NP: Nanoparticle(s)
PSL: Polystyrene latex
PTA: Particle tracking analysis
RI: Refractive index
SANS: Small-angle neutron scattering
SAXS: Small-angle X-ray scattering
SEM: Scanning electron microscopy
SOP: Standard operating procedure
spICP-MS: Single particle inductively coupled plasma mass spectrometry
TEM: Transmission electron microscopy
TOQ: Type of quantity
USSP: Ultrasonic spectroscopy
VSSA: Volume-specific surface area

## Symbols

*Q*_A_(*x*): Sum function weighted in quantity *A* (number like “3” refers to geometric properties, abbreviations like “ext” to physical properties)
*q*_A_(*x*): Density function weighted in quantity *A* (1/m)
*q*_A_^*^(*x*): Transformed density function in quantity *A*
*x*: Particle size, equivalent diameter (m)
*x*_BET_: BET equivalent diameter (i. e. computed from VSSA) (m)
*x*_BET,min_: BET equivalent minimum size (computed from VSSA assuming a certain type of particle shape) (m)
*x*_50,0_: Median size of the number-weighted size distribution (m)
*x*_Feret,min_: Minimum Feret diameter (=distance between parallel tangents) (m)
*x*_hd_: Hydrodynamic diameter (for equivalence to hydrodynamic drag) (m)
*x*_mob_: Mobility diameter (for equivalence to electric mobility of aerosol particles) (m)
*x*_p_: Diameter of constituent particles (m)
*x*_Stokes_: Stokes diameter (settling velocity equivalent diameter) (m)
x_V/S_: Volume/surface equivalent diameter (m)
