# Supplementary material for: How reliably can a material be classified as a nanomaterial? Available particle-sizing techniques at work
Source: J Nanopart Res. 2016 Jun 14;18:158. doi: 10.1007/s11051-016-3461-7 (PMC4908171; doi:10.1007/s11051-016-3461-7)
Supplement: Supplementary file 1 — Supplementary material 1 (PDF 1743 kb) [file 11051_2016_3461_MOESM1_ESM.pdf]

How reliably can a material be classified as a nanomaterial? Available particle sizing techniques at work

Frank Babick[a,*], Johannes Mielke[b], Wendel Wohlleben[c], Stefan Weigel[d] and Vasile-Dan Hodoroaba[b]

with contributions from: R. Bienert, S. Benemann, K. Brademann-Jock, M. Gemeinert, C. Kästner, F. Lindemann, G. Steinborn, A. Thünemann (BAM), U. Flörchinger, M. Kaiser, P. Müller, K. Werle, K. Vilsmeier, T. Wieczorek (BASF), M. Correia, K. Löschner (DTU), T. Mikael Uusimaeki (EAWAG), D. Mehn, F. Pianella (JRC-IHCP), R. Koeber, G. Roebben (JRC-IRMM), L. Coquelin, C. Motzkus (LNE), P. Vincent (Malvern), G. van Bemmel, C. Cascio, F. Gallocchio (RIKILT), L. Hillemann, A. Kupka, C. Ullmann (TUD), M. Velimirovic, S. Wagner (University of Vienna)

[a]*Technische Universität Dresden (TUD),*
Research Group of Mechanical Process Engineering, 01062 Dresden, GERMANY

[b]*Bundesanstalt für Materialforschung und -prüfung (BAM),*
Division 6.8 Surface analysis and interfacial chemistry, 12205 Berlin, GERMANY

[c]*BASF SE, Department of material physics, 67056 Ludwigshafen, GERMANY*

[d]*RIKILT – Wageningen UR, 6700 AE Wageningen, NETHERLANDS.*
(present: Bundesinstitut für Risikobewertung (BfR), 10589 Berlin, GERMANY)

[*] PD Dr.-Ing. habil. Frank Babick (corresponding author), Institut für Verfahrenstechnik und Umwelttechnik, Technische Universität Dresden, 01062 Dresden, Germany; phone: +49-351-46333724, fax: +49-351-46337058, e-mail: Frank.Babick@tu-dresden.de

Supplementary Material

S.1 Measurement reports and guidelines for ensuring high-level SOPs for conducting measurements

Measurement reports are accessible under:

https://cloudstore.zih.tu-dresden.de/public.php?service=files&t=df134ce4a94b90QCM4a3da0a16a050ba2b

(password: NanoDefine)

Guidelines are found under:

https://cloudstore.zih.tu-dresden.de/public.php?service=files&t=9b80518a1ee4a1b03112d67d93423e53

(password: NanoDefine)

S.2 Manual pre-treatment of measurement data

The data analysis could include a manual data treatment, which had to be declared in the measurement report and which should be in accordance to lab-internal standard procedures. There are three situations, when pre-treatment was considered justified: i) the removal of outliers from a series of measurements (e. g. when gas bubbles spoil the quality of the correlation function in DLS measurement), ii) the manual setting of the lower size limit, and iii) the manual setting of the maximum particle size. The lower limit of the size range needs to be manually adjusted for discAC-turb (where the steady baseline shift in addition to noise produces uncertainty with respect to the finest particle fractions), for spray-DEMA (where non-intentionally generated salt particles in the nano-range cannot be completely avoided), for AF4-techniques (where typically an initial "void peak" is observed before fractionation takes place), and for imaging techniques (when automated software algorithms erroneously identify debris from dispersion agents as particles). Note that some instruments may employ rules for defining the lower size limit within the data analysis software (e. g. threshold values for particles signals in spICP-MS). For some MTs it is sometimes necessary to define an upper limit of the size range. This holds true for DLS, where inversion algorithms may attribute all deviations between measured and ideally shaped correlation function to the coarsest size class, which generates a clearly separated, non-physical artefact mode. It also applies to AF4-techniques, where a release peak occurs, after the cross-field has been turned off.

S.3 Measurement instruments and partners involved

Table S-1 Measurement systems employed, their characteristic parameters during the analytical study and institutions where analyses were conducted

MT acronym	instrumentation	characteristic parameters (during measurement)	participant laboratory
TEM	STEM, HD 2700<br>FEI Tecnai G2	200 kV<br>200 kV	EAWAG<br>BASF
SEM	Zeiss Supra 40 (Zeiss, DE)	20 kV	BAM
spICP-MS	Nexion 350 (Perkin Elmer)		RIKILT
PTA	NS500, HS (Malvern)<br>Zetaview (Particle Metrix, DE)	405 nm<br>403 nm	Malvern<br>TUD
spray-DEMA	SMPS (TSI, USA)		LNE, TUD
discAC-turb	DQCM24000 UHR (CPS Instruments)	405 nm, 25000 g	JRC-IHCP
cuvAC-turb	LUMiSizer (LUM, DE)	470 nm, 300 g … 2250 g	TUD
cuvAC-RI	XLI Proteome Lab Version (Beckman, UK)	670 nm, 70 g … 27000 g	BASF
AF4-LS	Eclipse DUALTEC (S/N 4032), Wyatt Technology		UNIVIE
DLS	Zetasizer Nano ZS (Malvern, UK)<br>HPPS (Malvern, UK)	632 nm, 173°<br>632 nm, 173°	BAM<br>TUD
ALS	Mastersizer 2000 (Malvern, UK)<br>HELOS BR (Sympatec, DE)	632 nm & 470 nm, 0.2°…135°<br>632 nm, 0.5° … 37°	BAM<br>TUD
SAXS	SAXSess (Anton Paar, AUT)	Cu X-ray tube	BAM
USSP	each: DT 1200<br>(Dispersion Technol., USA)	3 MHz … 100 MHz<br>3 MHz … 100 MHz	BAM<br>TUD
BET	multi-point-BET with N_2 (ISO 9277)	77.3 K, p/p_0 = 0.001 …0.3	BAM, BASF

BAM: Bundesanstalt für Materialforschung und -prüfung, Division 6.8 Surface Analysis and Interfacial Chemistry (coordination of BAM activities), 12205 Berlin, GERMANY

BASF: BASF SE, Department of material physics, 67056 Ludwigshafen, GERMANY

EAWAG: Eidgenössische Anstalt für Wasserversorgung, Abwasserreinigung und Gewässerschutz, Particle laboratory, 8600 Dübendorf, SWITZERLAND

JRC-IHCP: Joint research centre – Institute of health and consumer protection, Nanobiosciences unit, 21027 Ispra (VA), ITALY

LNE: Laboratoire National de Metrologie et d'Essais, Paris, FRANCE

Malvern: Malvern Ltd, Amesbury, SP4 7RT, UNITED KINGDOM

RIKILT: RIKILT – Wageningen UR, 6700 AE Wageningen, NETHERLANDS

TUD: Technische Universität Dresden, Research group mechanical process engineering, 01062 Dresden, GERMANY

UNIVIE: Universität Wien, Department of environmental geosciences, 1090 Vienna, AUSTRIA

S.4 Values for skeleton density and refractive index

Table S-2 Skeleton density, and refractive index for several wavelengths employed by the optical MTs

code (material)	ρ, g/cm³	m_{405nm}	m_{470nm}	m_{530nm}	m_{633nm}	m_{670nm}	m_{865nm}
QCM1 (polystyrene)	1.050	1.624	1.608	1.598	1.587	1.584	1.576
QCM2 (colloidal SiO_2)	2.305	1.469	1.463	1.459	1.455	1.454	1.45
QCM3 (colloidal Au)	19.3	1.46-1.96i	1.28-1.88i	0.569-2.26i	0.155-3.36i	0.140-3.74i	0.175-5.48i
QCM4 (colloidal Ag)	10.5	0.170-2.03i	0.142-2.64i	0.140-3.15i	0.140-3.98i	0.140-4.27i	0.140-5.75i
QCM5 (3-mod PSL)	1.050	1.624	1.608	1.598	1.587	1.584	1.576
QCM6 (3-mod SiO_2)	2.305	1.469	1.463	1.459	1.455	1.454	1.45
RTM1 ($BaSO_4$, UF)	4.4	1.697	1.668	1.652	1.634	1.630	1.617
RTM2 ($BaSO_4$, fine)	4.4	1.697	1.668	1.652	1.634	1.630	1.617
RTM3 (coated TiO_2)	3.99	3.23	2.99	2.87	2.77	2.74	2.66
RTM4 ($CaCO_3$)	2.657	1.551	1.541	1.534	1.525	1.522	1.507
RTM5 (kaolin)	2.61				1.56		
RTM6 (fumed SiO_2)	2.2	1.469	1.463	1.459	1.455	1.454	1.45
RTM7 (pigment Y83)	1.484	1.47-0.457i	1.92-0.42i	1.93-0.07i	1.75-0.029i	1.73-0.023i	1.72-0.03i
RTM8 (pigment Y83)	1.5	1.47-0.457i	1.92-0.42i	1.93-0.07i	1.75-0.029i	1.73-0.023i	1.72-0.03i
RTM9 (methacrylate)	1.13	1.391	1.387	1.384	1.381	1.381	1.378
water (H_2O)	0.997	1.343	1.338	1.335	1.332	1.331	1.328

The extent to which the values of material properties affect the measured particle size (distribution) may be weak for one particle system and rather huge for others; they can affect the measured particle size or "just" the conversion to Q_0. In some case it is possible to quantify the relative uncertainty $U_x = |\Delta x/x|$ of measured size (x) as function of the relative uncertainty in material properties like density (ρ):

- spICP-MS: impact of density on size $U_x = \frac{1}{3} U_\rho$
- mobility based MTs (PTA, AF4-LS, AC techniques, DLS): reciprocal impact of liquid viscosity (η) on size $U_x = U_\eta$
- AC techniques: impact of density contrast ($\Delta\rho$) on size $U_x = \frac{1}{2} U_{\Delta\rho}$
- conversion from Q_{ext} to Q_0 or from Q_{int} to Q_0 (AF4-LS, AC techniques, DLS): nonlinear impact of RI

- ALS: nonlinear impact of RI on size distribution
- USSP: nonlinear impact of density contrast ($\Delta\rho$) and liquid viscosity (η) on size distribution
- BET: reciprocal impact of density on size $U_x = U_\rho$

Only few MTs determine the particle size without the need to account for material properties (TEM, SEM, SAXS).

Details on the wavelength dependency of refractive index data including references are available under

https://cloudstore.zih.tu-dresden.de/public.php?service=files&t=9b80518a1ee4a1b03112d67d93423e53

(password: NanoDefine).

S.5 <u>SEM images of quality control materials</u>

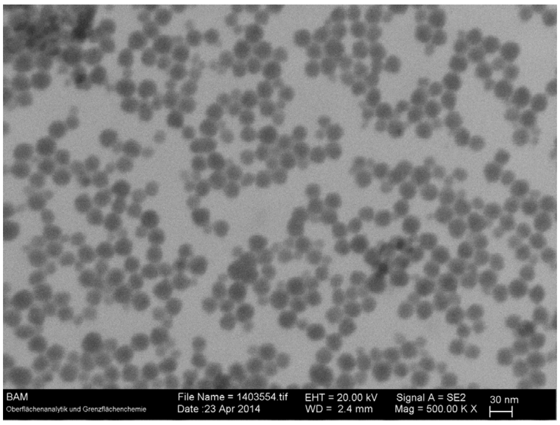

Fig. S-1 SEM image of QCM2: nano-sized SiO2

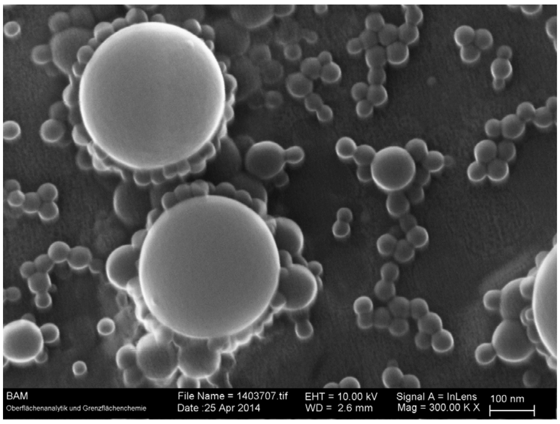

Fig. S-2 SEM image of QCM5: trimodal PSL

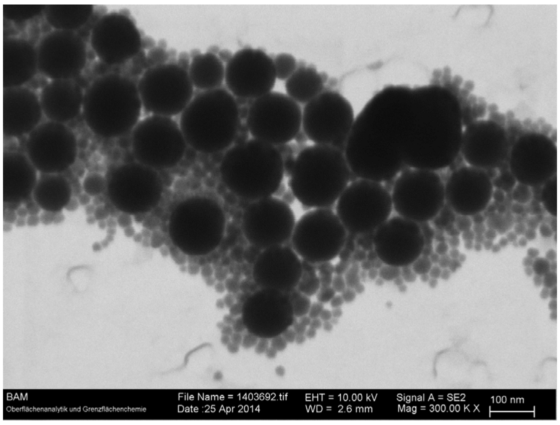

Fig. S-3 SEM image of QCM6 (3mod SiO2)

S.6 Graphical representation of number weighted size distributions

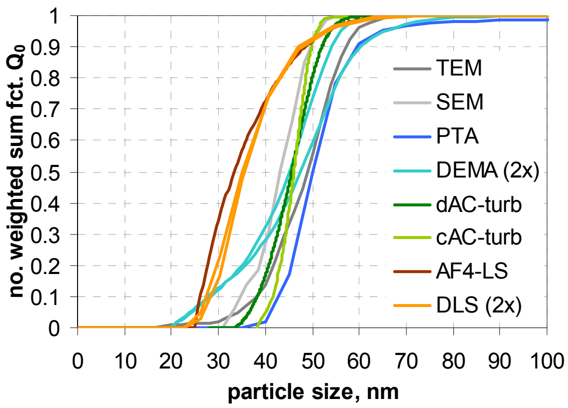

Fig. S-4 QCM1 (monomodal polystyrene), number weighted sum functions from measurements with TEM, SEM, PTA, spray-DEMA (2×), discAC-turb, cuvAC-turb, cuvAC-RI, AF4-LS, and DLS (2×)

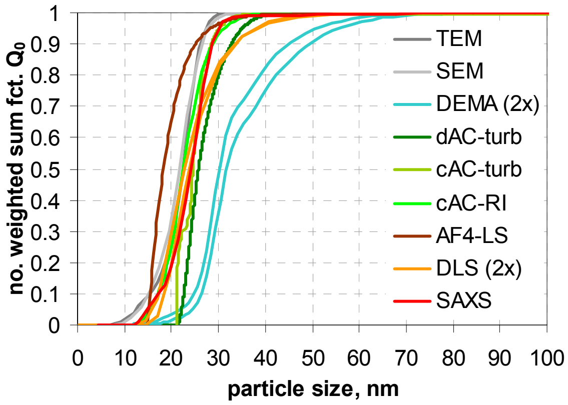

Fig. S-5 QCM2 (colloidal SiO_2), number weighted sum functions from measurements with TEM, SEM, spray-DEMA (2×), discAC-turb, cuvAC-turb, cuvAC-RI, AF4-LS, DLS (2×), and SAXS

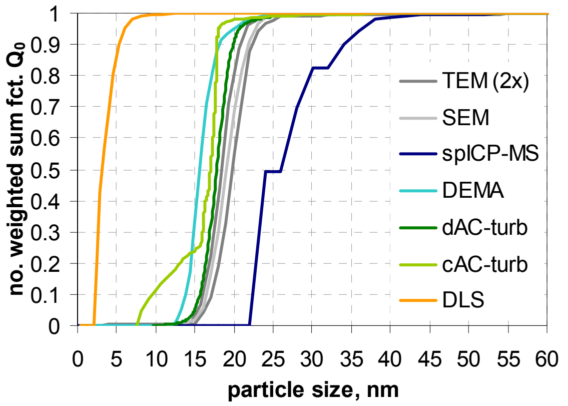

Fig. S-6 QCM3 (colloidal Au), number weighted sum functions from measurements with TEM (2×), SEM, spICP-MS, spray-DEMA, discAC-turb, cuvAC-turb, and DLS

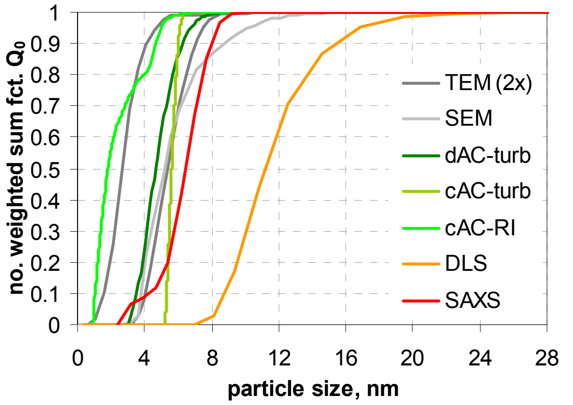

Fig. S-7 QCM4 (colloidal Ag), number weighted sum functions from measurements with TEM (2×), SEM, discAC-turb, cuvAC-turb, cuvAC-RI, DLS and SAXS

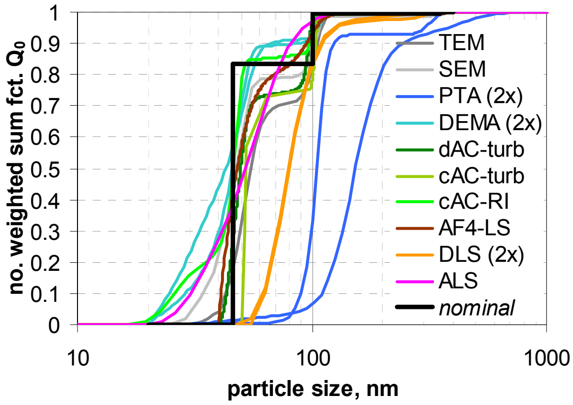

Fig. S-8 QCM5 (trimodal polystyrene), number weighted sum functions from measurements with TEM, SEM, PTA (2×), spray-DEMA (2×), discAC-turb, cuvAC-turb, cuvAC-RI, AF4-LS, DLS (2×), and ALS

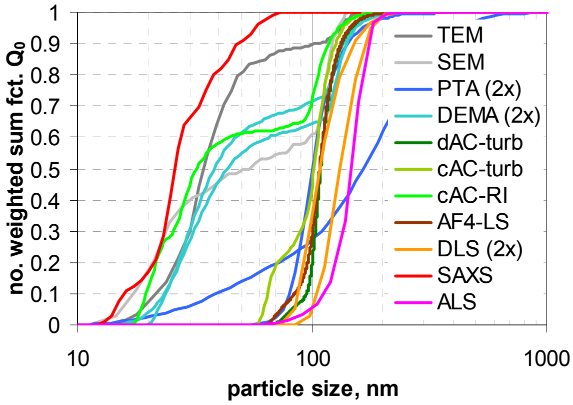

Fig. S-9 QCM6 (trimodal SiO_2), number weighted sum functions from measurements with TEM, SEM, PTA (2×), spray-DEMA (2×), discAC-turb, cuvAC-turb, cuvAC-RI, AF4-LS, DLS (2×), SAXS, and ALS

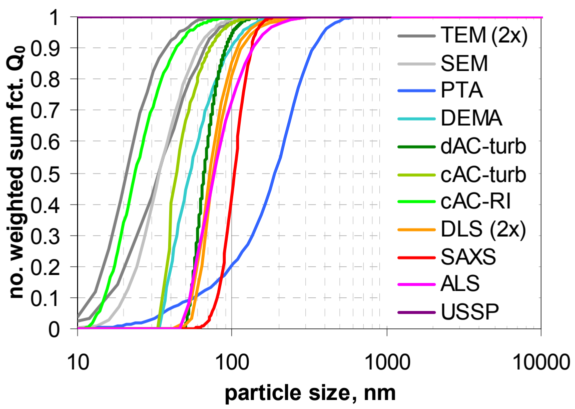

Fig. S-10 RTM1 ($BaSO_4$, ultrafine), number weighted sum functions from measurements with TEM (2×), SEM, PTA, spray-DEMA, discAC-turb, cuvAC-turb, cuvAC-RI, DLS (2×), SAXS, ALS, and USSP

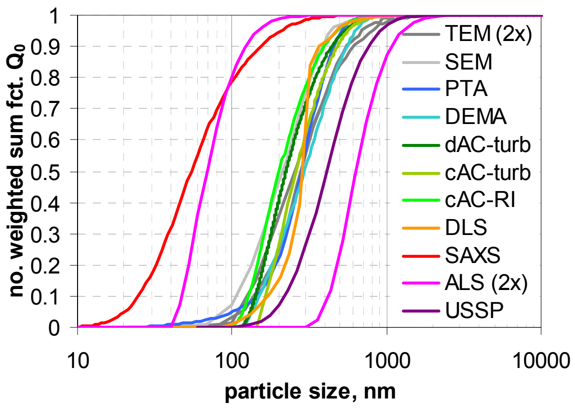

Fig. S-11 RTM2 ($BaSO_4$, fine), number weighted sum functions from measurements with TEM (2×), SEM, PTA, spray-DEMA, discAC-turb, cuvAC-turb, cuvAC-RI, DLS, SAXS, ALS (2×), and USSP

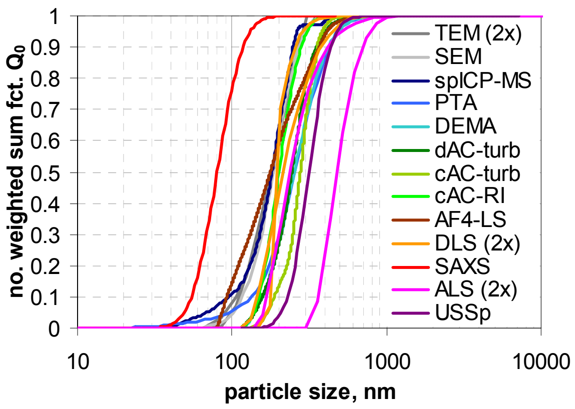

Fig. S-12 RTM3 (coated TiO_2), number weighted sum functions from measurements with TEM (2×), SEM, spICP-MS, PTA, spray-DEMA, discAC-turb, cuvAC-turb, cuvAC-RI, DLS (2×), SAXS, ALS (2×), and USSP

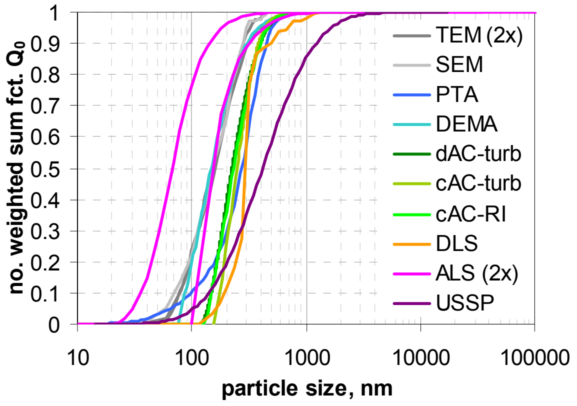

Fig. S-13 RTM4 ($CaCO_3$), number weighted sum functions from measurements with TEM (2×), SEM, PTA, spray-DEMA, discAC-turb, cuvAC-turb, cuvAC-RI, ALS (2×), and USSP

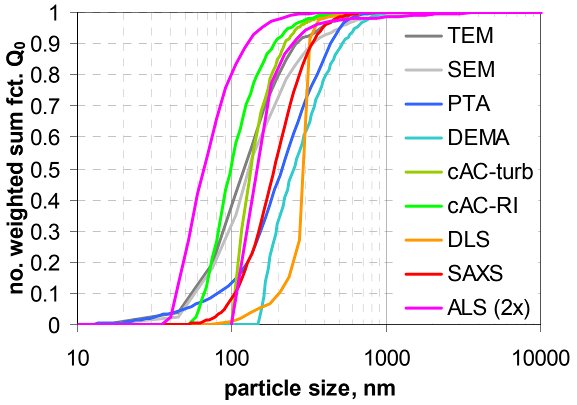

Fig. S-14 RTM5 (kaolin), number weighted sum functions from measurements with TEM, SEM, PTA, spray-DEMA, cuvAC-turb, cuvAC-RI, DLS, SAXS, and ALS (2×)

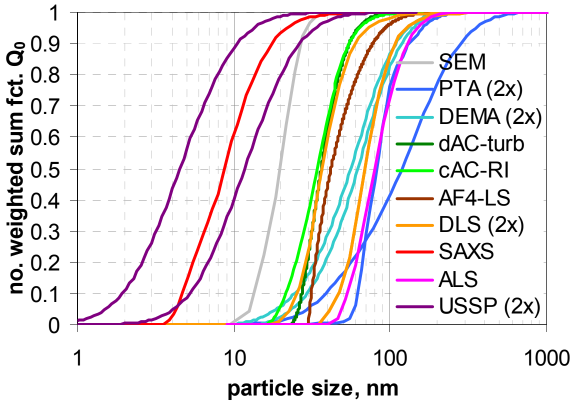

Fig. S-15 RTM6 (fumed SiO_2), number weighted sum functions from measurements with SEM, PTA (2×), spray-DEMA (2×), discAC-turb, cuvAC-RI, AF4-LS, DLS (2×), SAXS, ALS, and USSP (2×)

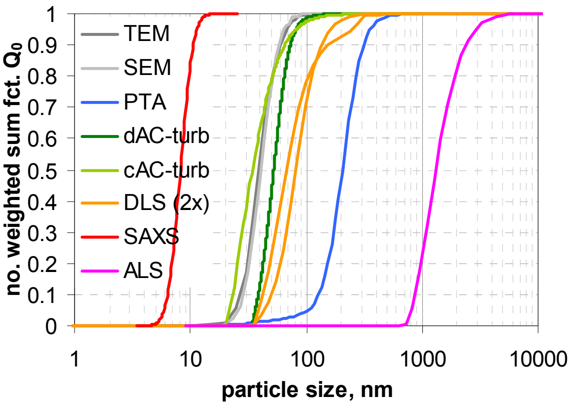

Fig. S-16 RTM7 (pigment Y83, transparent), number weighted sum functions from measurements with TEM, SEM, PTA, discAC-turb, cuvAC-turb, DLS (2×), SAXS, and ALS

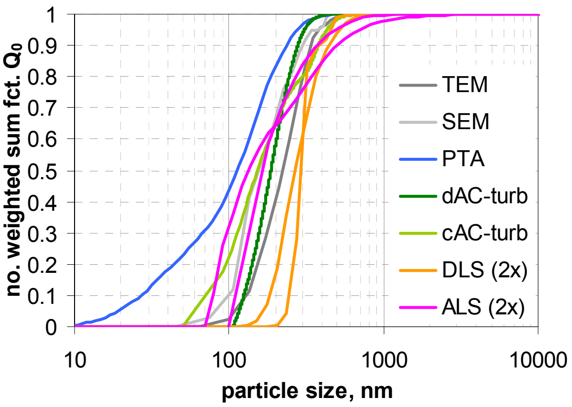

Fig. S-17 RTM8 (pigment Y83, opaque), number weighted sum functions from measurements with TEM, SEM, PTA, discAC-turb, cuvAC-turb, DLS (2×), and ALS (2×)

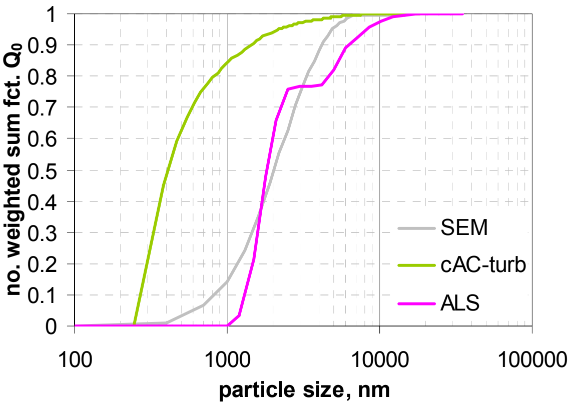

Fig. S-18 RTM9 (BMC), number weighted sum functions from measurements with SEM, cuvAC-turb, and ALS

S.7 Volume weighted size distributions

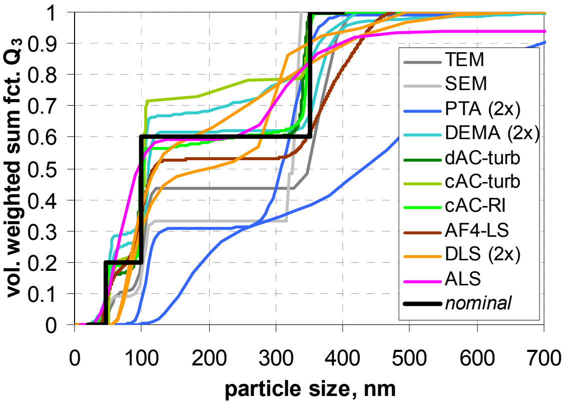

Fig. S-19 QCM5 (trimodal PSL) volume weighted sum functions from measurements with TEM, SEM, PTA (2×), spray-DEMA (2×), discAC-turb, cuvAC-turb, cuvAC-RI, AF4-LS, DLS (2×) and ALS

S.8 Discussion on the quality of measurement data

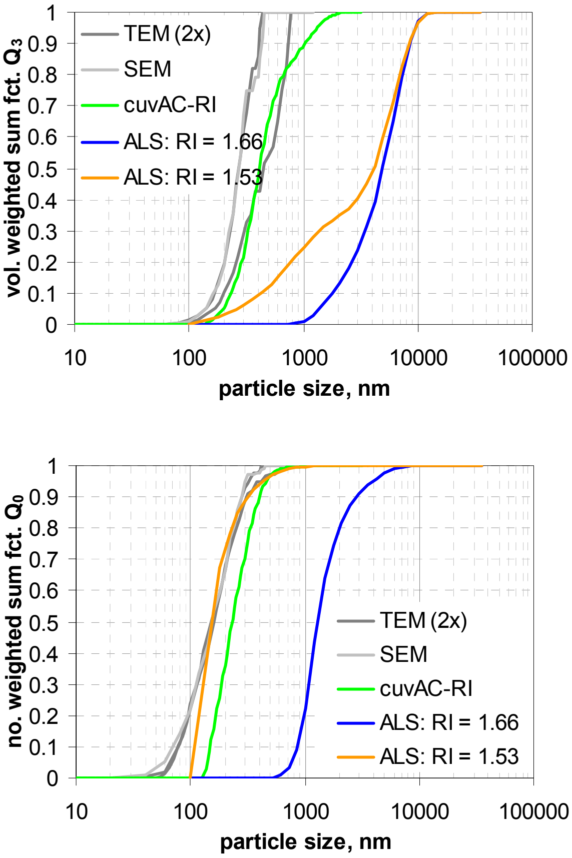

a)

b)

Fig. S-20 ALS result for RTM4, impact of RI values on a) volume and b) number weighted size distribution (additionally results of TEM, SEM & cuvAC-RI)

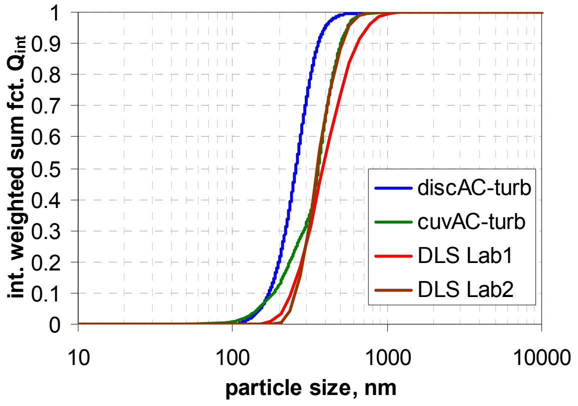

a)

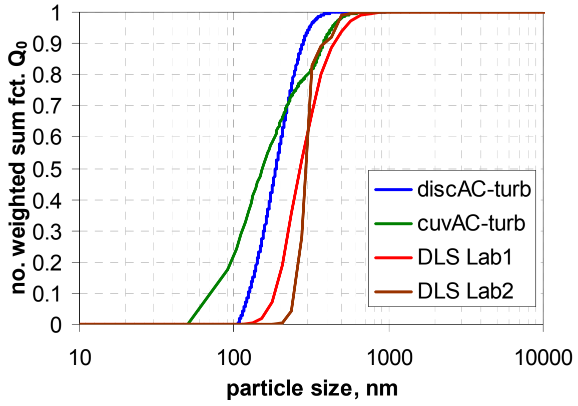

b)

Fig. S-21 RTM8 (PY83, opaque) results of mobility-based MTs with particle quantification by light extinction or light scattering; a) intrinsically measured intensity and extinction weighted and b) derived number weighted sum functions

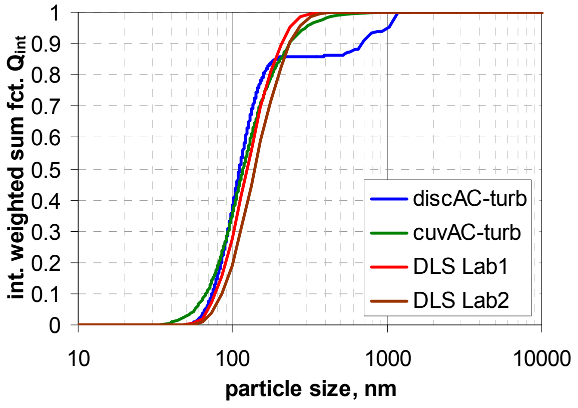

a)

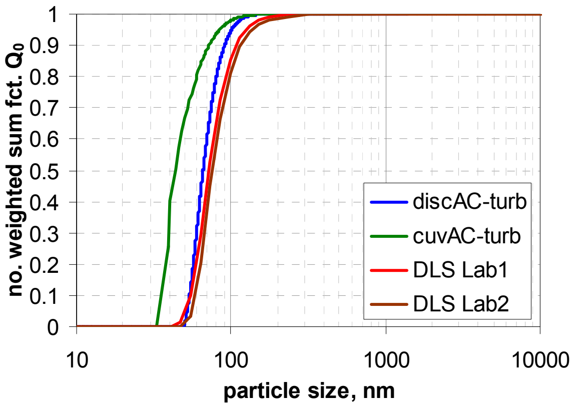

b)

Fig. S-22 RTM1 ($BaSO_4$, ultrafine) results of mobility-based MTs with particle quantification by light extinction or light scattering; a) intrinsically measured intensity or extinction weighted and b) derived number weighted sum functions

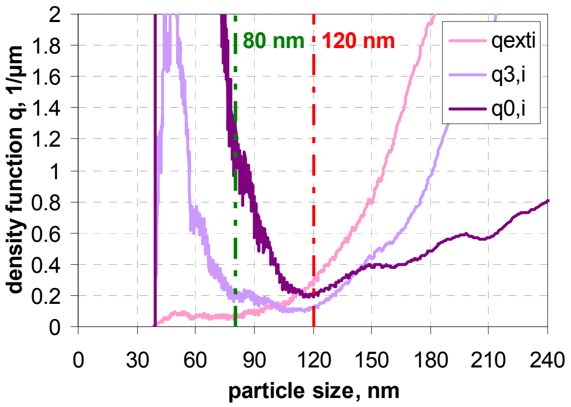

a)

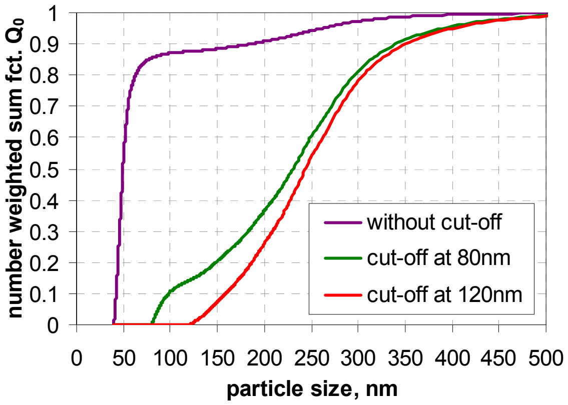

b)

Fig. S-23 impact of setting a lower size limit on Q_0 for RTM3 (coated TiO_2) when measured with discAC-turb; a) detail of density functions for unbound size range and possible cut-off values for the lower size limit, b) sum functions of number weighted size distribution for different values of the lower size limit

S.9 Parity plots with indication of intermediate precision

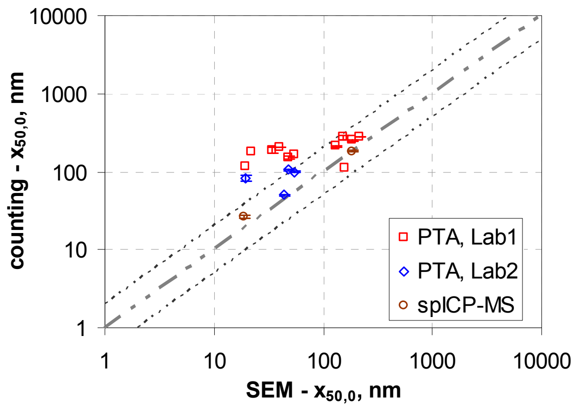

a)

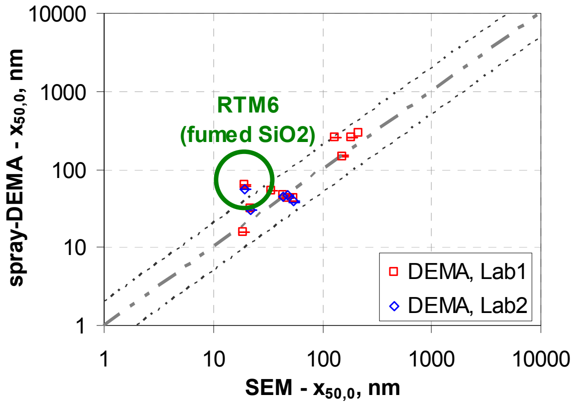

b)

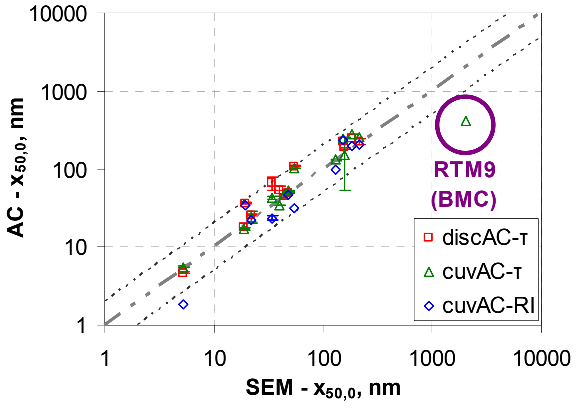

c)

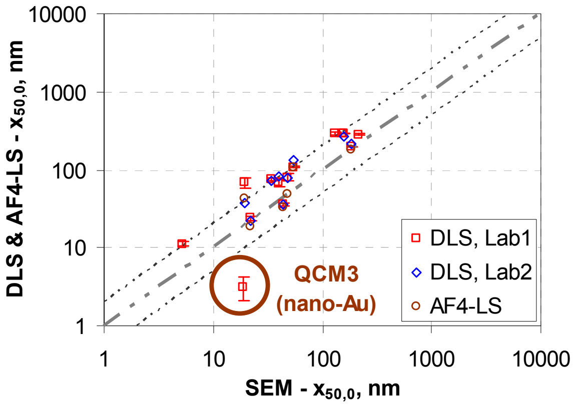

d)

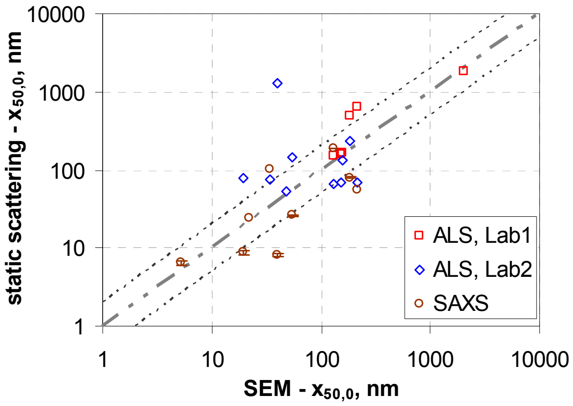

e)

Fig. S-24 Parity plots of the number weighted medians $x_{50,0}$ as determined by the various MTs vs. the SEM value (cf. Table 4), lines indicate parity and deviation from parity by a factor of 2, error bars refer to intermediate precision as reported in Table 6; a) non-imaging counting techniques (PTA, spICP-MS), b) fractionating techniques with a counting detector (spray-DEMA), c) AC techniques (discAC-turb, cuvAC-turb, cuvAC-RI), d) mobility-based techniques with a light scattering detector (DLS, AF4-LS), e) static scattering techniques (ALS, SAXS)

S.10 Additional data on TEM and SEM results

Table S-3 Number of particles employed in size analysis with EM techniques and degree of polydispersity indicated as ratio of 99 %-quantile to 1 %-quantile form SEM analysis (in brackets: ratio of maximum to minimum size)

code (material)	N(SEM)	N(TEM-1)	N(TEM-2)	$x_{99,0}/x_{1,0}$ (x_{max}/x_{min})
QCM1 (polystyrene)	65	923		1.7 (1.8)
QCM2 (colloidal SiO_2)	923	1666		3.0 (5.2)
QCM3 (colloidal Au)	1748	899	339	1.7 (2.4)
QCM4 (colloidal Ag)	2133	4177	388	3.8 (54)
QCM5 (3-mod PSL)	251	872		12 (14)
QCM6 (3-mod SiO_2)	147	466		10 (13)
RTM1 ($BaSO_4$, UF)	5816	259	691	7.1 (21)
RTM2 ($BaSO_4$, fine)	234	1633	372	8.5 (16)
RTM3 (coated TiO_2)	9456	319	58	4.5 (16)
RTM4 ($CaCO_3$)	133	261	327	8.7 (15)
RTM5 (kaolin)	753			24 (90)
RTM6 (fumed SiO_2)	147			2.9 (3.6)
RTM7 (pigment Y83)	129	366		3.5 (4.6)
RTM8 (pigment Y83)	75	353	225	6.1 (7.2)
RTM9 (methacrylate)	390			14 (40)

S.11 Glossary

particle size x

equivalent diameter or characteristic length

equivalent diameter x_Z

diameter of a sphere being equivalent to particle with respect to property Z

characteristic length

any length describing the outer dimensions of a particle (mostly in image analysis), e. g. minimum Feret diameter, rod diameter, cord length

measurement principle

(physical) effect that is employed for the quantification of the measurand (e. g. particle size, zeta-potential); the dependency of the effect on the measurand has to be well-defined; examples: i) settling velocity grows with particle size, ii) diffusion coefficient is inversely proportional to size, iii) 2D-images obtained by orthogonal projection allow the direct quantification of characteristic lengths and areas.

measurement technique (MT)

specific way of employing a measurement principle for the quantification of a measurand (e. g. particle size); similar measurement techniques are sometimes grouped as one measurement method; examples: i) the settling velocity as a measure of size (= measurement principle) can be measured by gravitational sedimentation with an X-ray transmission detector (= measurement technique) or by centrifugal sedimentation in homogenously filled cuvettes with an optical transmission detector, ii) the diffusion coefficient can be measured with dynamic light scattering by means of a self-beating autocorrelation detector or by means of homodyne light detection and frequency analysis, iii) sphere diameters can be derived from images of a scanning electron microscope, which were obtained with or without using the emitted secondary electrons.

characterisation method (CM)

specific procedure for the characterisation of a material which includes the steps of sampling, sample preparation, sample analysis with (a) certain measurement technique(s) and defined algorithms of signal interpretation, and final data interpretation

quality control material (QCM) for particle size analysis

a particulate material relatively well defined with respect to particle shape (spherical or similar), state of aggregation (non-aggregated, non-agglomerated) and particle size distribution, which are supplied as ready-to-use suspension samples; cf. ISO Guide 30:2015

representative test materials (RTM) for particle size analysis

a particulate material from a single batch, which is sufficiently homogeneous and stable with respect to one or more properties that affect particle size analysis; can be provided as powder or suspension and requires defined

sample preparation (e. g. to obtain sufficiently stable, well-dispersed suspension samples); cf. ISO TS 16195:2013
